# Unleashing the promise of emerging nanomaterials as a sustainable platform to mitigate antimicrobial resistance

**DOI:** 10.1039/d3ra05816f

**Published:** 2024-05-01

**Authors:** Sazedur Rahman, Somya Sadaf, Md Enamul Hoque, Akash Mishra, Nabisab Mujawar Mubarak, Guilherme Malafaia, Jagpreet Singh

**Affiliations:** a Department of Mechanical and Production Engineering, Ahsanullah University of Science and Technology Dhaka Bangladesh; b Department of Civil and Environmental Engineering, Birla Institute of Technology Mesra Ranchi 835215 Jharkhand India; c Department of Biomedical Engineering, Military Institute of Science and Technology Dhaka Bangladesh enamul1973@gmail.com; d Petroleum and Chemical Engineering, Faculty of Engineering, Universiti Teknologi Brunei Bandar Seri Begawan BE1410 Brunei Darussalam; e Department of Chemistry, School of Chemical Engineering and Physical Sciences, Lovely Professional University Jalandhar Punjab India; f Post-Graduation Program in Conservation of Cerrado Natural Resources, Goiano Federal Institute Urutaí GO Brazil; g Department of Chemistry, University Centre for Research and Development, Chandigarh University Mohali-140413 India jagpreet.e12147@cumail.in jagpreetnano@gmail.com

## Abstract

The emergence and spread of antibiotic-resistant (AR) bacterial strains and biofilm-associated diseases have heightened concerns about exploring alternative bactericidal methods. The WHO estimates that at least 700 000 deaths yearly are attributable to antimicrobial resistance, and that number could increase to 10 million annual deaths by 2050 if appropriate measures are not taken. Therefore, the increasing threat of AR bacteria and biofilm-related infections has created an urgent demand for scientific research to identify novel antimicrobial therapies. Nanomaterials (NMs) have emerged as a promising alternative due to their unique physicochemical properties, and ongoing research holds great promise for developing effective NMs-based treatments for bacterial and viral infections. This review aims to provide an in-depth analysis of NMs based mechanisms combat bacterial infections, particularly those caused by acquired antibiotic resistance. Furthermore, this review examines NMs design features and attributes that can be optimized to enhance their efficacy as antimicrobial agents. In addition, plant-based NMs have emerged as promising alternatives to traditional antibiotics for treating multidrug-resistant bacterial infections due to their reduced toxicity compared to other NMs. The potential of plant mediated NMs for preventing AR is also discussed. Overall, this review emphasizes the importance of understanding the properties and mechanisms of NMs for the development of effective strategies against antibiotic-resistant bacteria.

## Abstract 2

The emergence and spread of antibiotic-resistant (AR) bacterial strains and biofilm-associated diseases have heightened concerns about exploring alternative bactericidal methods.

## Graphical abstract

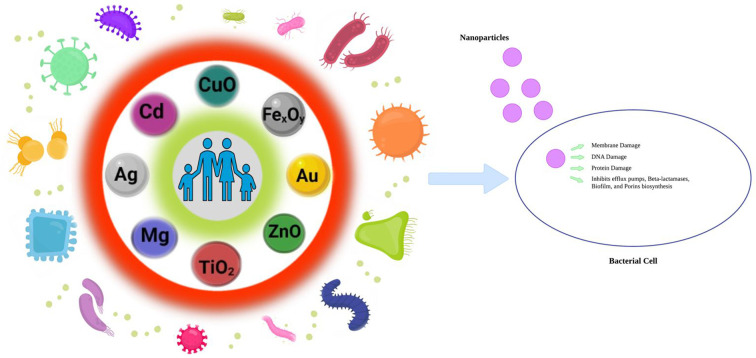

## 1. Introduction

**Fig. 1:**
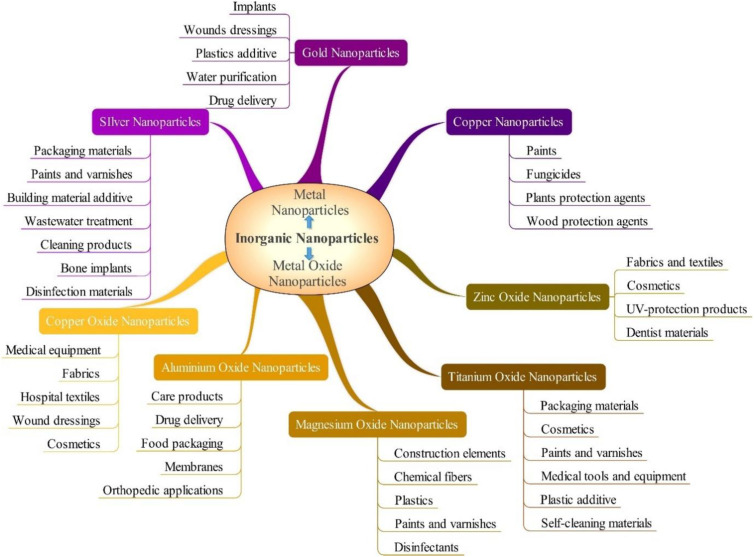
Antimicrobial inorganic nanoparticles and their uses (Reprinted from^21^ by permission of the Taylor & Francis Ltd, https://www.tandfonline.com/).

Nanotechnology emerged as a crucial area of scientific research since the 21st century, and drives technological advancements in computer chips, construction materials, healthcare, environmental protection, new energy forms, and electricity transportation.^1–4^ Nanomaterials are materials having specific physical or chemical characteristics or biological impacts that have an exterior size, internal size, or surface structure that falls within the nanoscale range.^5–7^ More precisely, a nanostructured material is thought to have dimensions ranging from 1 to 100 nm.^8–10^ However, in medicine, these values may vary up to a diameter of 200 nm.^11^ In the context of the medication delivery system, nanotechnology is an inventive method that can be utilized. Currently, a diverse range of natural and synthetic nanomaterials are being researched to determine whether they can be used as medication delivery systems, as the rise of drug-resistant strains of disease-causing bacteria is one of human health's most significant concerns today. More than 2 million serious diseases are caused by antibiotic-resistant bacteria globally.^12,13^ Recent research shows that by the year 2050, bacterial infections are expected to cause 10 million deaths annually, which will exceed the current number of deaths caused by cancer.^14^ Therefore, nanomaterials are used as new antimicrobial agents to treat and prevent infectious diseases. Nanomaterials can overcome antibiotic resistance mechanisms by executing various new bactericidal routes, binding and breaking bacterial membranes, and causing cytoplasmic component leakage because of their unique physio-chemical characteristics.^15^ Nanomaterials can target biofilms and overcome recalcitrant infections due to their size (average diameter of 100 nm) and physical properties (size, shape, surface area, composition, *etc.*).^16^ In addition, they are effective against bacteria resistant to antibiotics because of their large surface-to-volume ratios, which make the surface-to-inner atom ratio of the material higher than in bulk materials with the same surface-to-volume proportions.^17–19^ Thus, nanomaterial-based therapeutic approaches are promising tools for combating difficult-to-treat bacterial infections, as they can circumvent existing mechanisms affiliated with multi-drug resistance.^20^ So, it is worthwhile to write a review on the investigation of nanomaterials and their efficacy against antibiotic resistance. Fig. 1 demonstrates the classification of inorganic nanoparticles with antibacterial characteristics and their uses.

This article comprehensively analyzes the significance of nanomaterials in addressing antibiotic resistance by elaborately discussing every related aspect. For different types of nanomaterials, the review discusses the techniques of interacting with microorganisms and the mechanism to fight against microbial pathogens. In addition, the physicochemical features of nanomaterials are presented in this review to study their toxicity so that health and safety can be ensured for nanomaterial usage. This review also presents economical and environmentally friendly plant-based nanomaterial extraction and the efficacy of nanomaterials as a novel drug delivery system. Furthermore, this review suggests that nanomaterials hold great promise for revolutionizing the field of medical science through their ability to mitigate antimicrobial resistance in the human body and their potential to replace traditional chemical antibiotics.

### 1.1. Antibiotic resistance

Antimicrobial resistance in pathogenic bacteria is a chronic global health concern affecting every region of the world and is linked with high morbidity and death rates.^22–27^ Recently, antibiotics have been misused and overused, allowing bacteria to proliferate and increasing antimicrobial resistance (AMR).^28^ This has resulted in antibiotics losing their power to halt the spread of illnesses and has resulted in a rise in the prevalence of infectious diseases.^29–32^ Convincing data connects antibiotic consumption to the emergence of resistance. Although guidelines and restrictions influence consumption, it is ultimately the result of several physician consultations.^33–35^ Antibiotic resistance is a natural phenomenon that arises in bacteria because of the inherent evolutionary character of bacteria, as well as bacteria's facile and quick adaption to different environments, which has both microbiological and clinical definitions.^36^ Microbiologically, antibiotic resistance can be defined as the existence of a genetically specified resistance mechanism that has been acquired or mutated, which classifies the microorganism as resistant or vulnerable depending on using a specified break in a clinical laboratory test. In contrast, “clinical resistance” refers to antimicrobial activity correlated with an increased likelihood of unsuccessful treatment.^37^

**Fig. 2:**
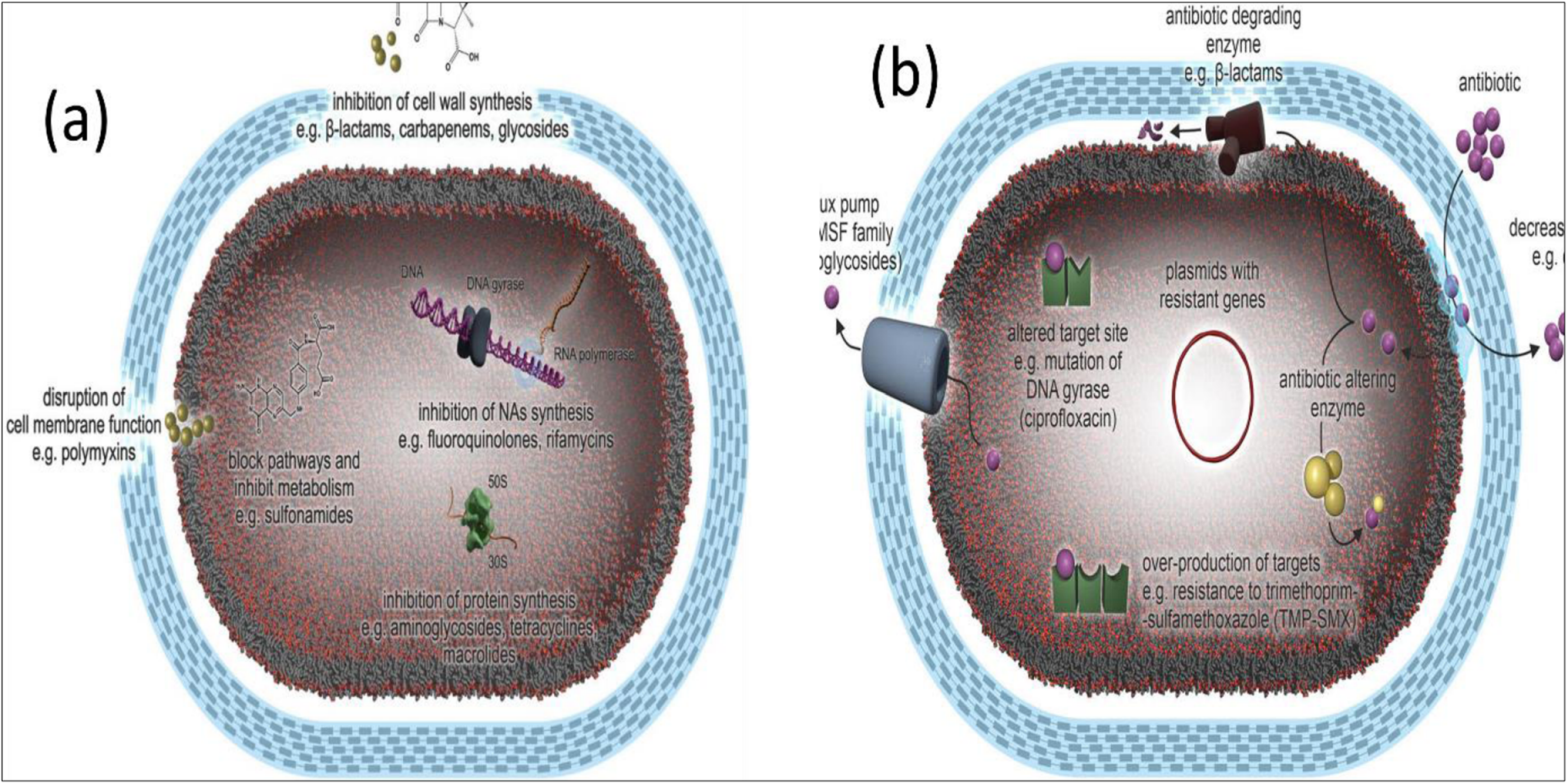
(a) Antibiotic mechanism of action, and (b) antibiotic resistance mechanisms in bacteria (Reprinted with permission from,^40^ An Open Access article distributed under the terms of the Creative Commons Attribution License).

In most cases, the mechanism of action of an individual antibiotic is sufficient enough to allow it to operate against only one particular target spot inside the bacterial cell. Antibiotics are known for their ability to impede bacterial cell wall formation, disrupt the cellular membranes, inhibit nucleic acid synthesis, inhibit photosynthesis, and inhibit folic acid synthesis, as illustrated in Fig. 2a.^38^ Several stages are required for an antibiotic to exert its antibacterial effect. It must first penetrate bacterial cells (influx), then be stable or be triggered and collected to inhibitory amounts. After that, it can find its target, engage with it, and exert antimicrobial effects. Regardless of the antibiotic's mechanism of action, chemical composition, or range of activity, changes to either of these stages lead to bacterial resistance.^39^

The resistance mechanism to antibacterial drugs is demonstrated in Fig. 2b. Antibacterial resistance mechanisms may be categorized as follows.^41^

✓ The development of enzymatic action that interferes with or alters the structure of antimicrobials,

✓ Alterations in the permeability of the bacterial wall and the membrane surrounding the cytoplasm,

✓ Changes made to the target locations of antibacterial agents,

✓ Enhanced removal of an antibiotic from the cells of bacteria (bacterial efflux).

**Fig. 3:**
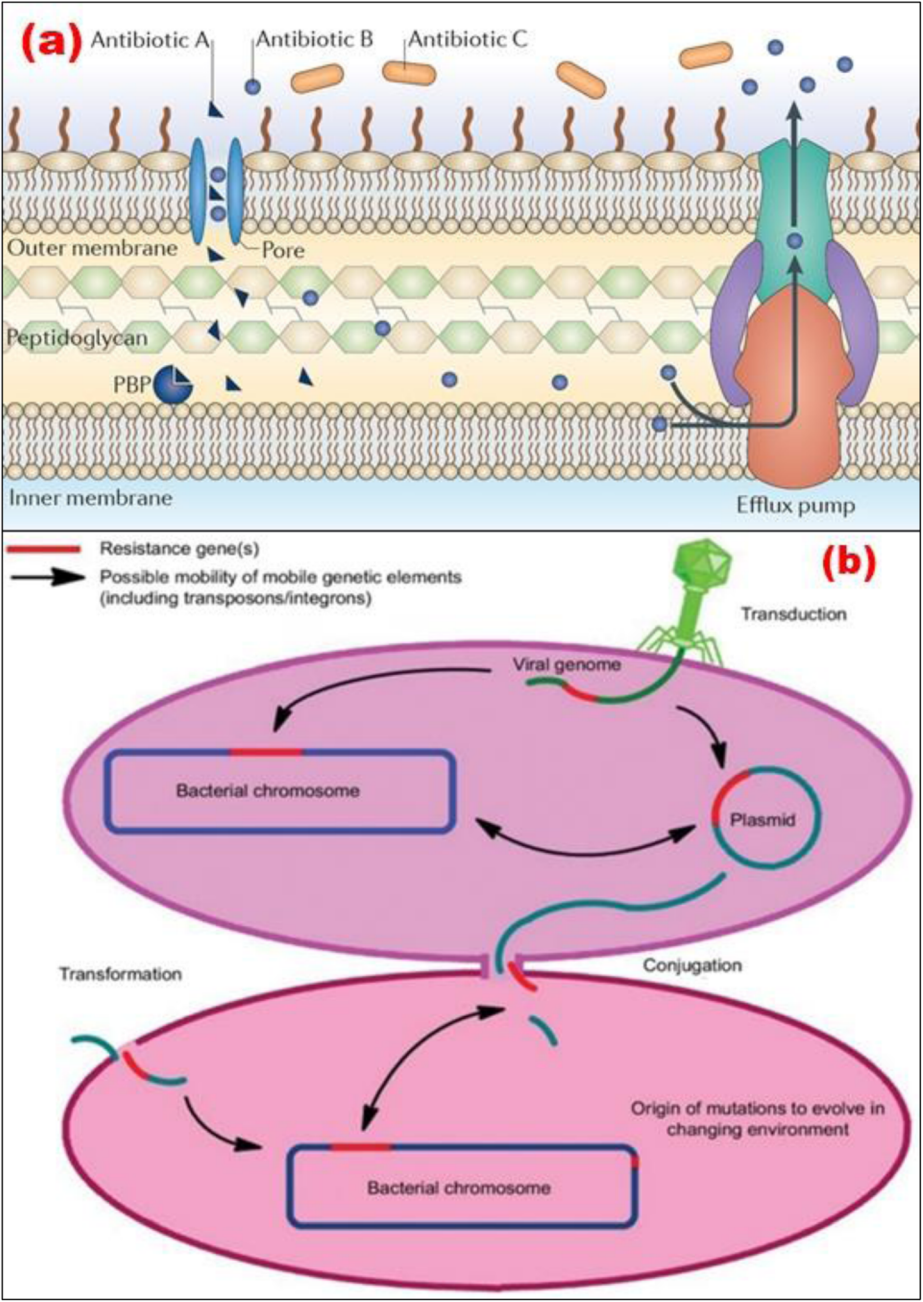
(a) Intrinsic antibiotic resistance mechanisms (Reprinted by permission from Springer Nature Customer Service Centre GmbH: Springer,^42^ Copyright 2015); (b) transmission factors for antimicrobial resistance (Reprinted with permission from,^43^ An Open Access article distributed under the terms of the Creative Commons Attribution License).

Therefore, the ability of a species of bacteria to withstand the activity of a certain antibiotic owing to inherent structural and functional characteristics can be defined as the resistance mechanism for that species of bacteria. Fig. 3a illustrates how β-lactam antibiotics may target a Penicillin-binding protein (PBP) to kill bacteria. In this instance, as shown in Fig. 3a, antibiotic A can enter the cell through a protein that spans the membrane, travel to its target, and then prevent the formation of peptidoglycan when it has been there. Further, Antibiotic B can also enter the cell through a porin, just like Antibiotic A. However, unlike Antibiotic A, Antibiotic B is effectively eliminated *via* efflux. Antibiotic C cannot get through the outer membrane, so it cannot enter the PBP, which is the target of its action. Hence, the antibiotics fail to kill the target bacteria.^42^

Several investigations have demonstrated that reasons behind the treatment, selection of agent, and duration of antibiotic treatment are all unsuitable 30–50% of the time.^43–45^ Antibiotic resistance has several causes, including insufficient regulatory requirements and usage inconsistencies, a lack of awareness of best practices that lead to unnecessary or ineffectual antibiotic usage, feeding antibiotics rather than regulating infection in poultry and livestock, and internet marketing made inexpensive antibiotics readily available.^46,47^ The development of antibiotic resistance is a natural process that occurs in bacteria, as illustrated in Fig. 3b. However, using different antibiotics in agriculture, healthcare, and the environment is responsible for antibiotic resistance. Additional major elements that are dominant factors for antibiotic resistance include infection control guidelines, water hygiene systems, medicine quality, sanitation settings, diagnostics and therapies, and movement restrictions.^43^ Moreover, the transfer of genes across organisms, in addition to changes in several genes upon that microorganism's chromosome, plays a significant part in the development of antibiotic resistance.^48^

There are two distinct ways in which the AMR gene may be transferred: chromosomal mutation and extrachromosomal mutation. Chromosome mutation situations arise when alterations are formed in the entire genome of bacteria, especially on the main chromosome, and it is shown through transmissions along the vertical direction, which means that it is passed down through generations of progeny. Chromosomal mutation occurs on its own and cannot be reversed. It causes alterations in the bacterial genome because of various aspects that can be physical, chemical, or both. These changes, in turn, cause changes inside the microorganism that modify the permeability and the drug objective to prevent antibiotics from affecting bacteria.^49^ As a result, chromosomal mutation relies on whether the pathogen's appropriateness or virulence changes. If genetically changed microorganisms dominate or develop more often, they would begin to reproduce and cause disorders.

Furthermore, another type of antimicrobial gene transfer is extrachromosomal resistance, which can be characterized as the transmission of genetic material through plasmids, integrons, and transposons. Plasmid transmission may transmit antibiotic-resistance genes to the host cell, and drugs can alter this process by increasing transmission.^49^ On the contrary, transposons are sequences already present in the genome and have a high capacity for recombination and mobility. This implies that they can readily be incorporated into a bacterium's genome. It is possible to move them from one plasmid to another and from a plasmid toward a chromosome *vice versa*.^50,51^ In addition, in the event of integrons capable of encoding AMR cassettes skilled in catching and expressing integrase genes, such integrons recognize the foreign gene and integrate it at integron sites.

Moreover, bacteria can convey resistance genes to other bacteria *via* horizontal gene transfer, which increases the potential for this phenomenon to spread.^58^ In addition, bacteria that develop resistance to numerous medications are known as multi-drug resistant bacteria (MDR) and are sometimes known colloquially as “superbugs.” Any bacterium has the potential to acquire multi-drug resistance (MDR). Nonetheless, a category of pathogens known as the “ESKAPE” group has a heightened propensity to do so. The “ESKAPE” group consists of the individuals listed below:

✓ E means *Enterococcus faecium*,

✓ S means *Staphylococcus aureus*,

✓ K means *Klebsiella pneumoniae*,

✓ A means *Acinetobacter baumannii*,

✓ P means *Pseudomonas aeruginosa*, and.

✓ E means *Enterobacter* spp.

### 1.2. Importance of nanomaterials in AMR

Nanotechnology is now essential in scientific and technical advancements in the medical and pharmaceutical fields. Nanomaterials enable altering materials' chemical and physical characteristics.^52,53^ In addition, the bigger the surface-volume ratio of the nanoparticles, the stronger their chemical and biological activity.^54^ Nanomaterials have been used for various applications, notably drug delivery, light ablation treatment, biological imaging, biosensor applications, and even as an approach to prevent antibiotic resistance, and have shown to be highly advantageous.^55,56^ Thus, some nanomaterials have features that make them effective against viruses, bacteria, and fungi, and they also have a high capability for combating illnesses caused by pathogens.^57,58^

Nanomaterials as antimicrobial supplements to antibiotics are very promising and receiving widespread attention because they can fill voids where medicines typically fail, such as battling mutants resistant to many drugs and biofilm.^59,60^ Nanomaterials, such as organic nanoparticles and metal oxide, now used in antimicrobial therapy, exhibit various inherent and changed chemical composition characteristics. Every one of the nanoparticles, independent of the chemical makeup, can combat bacteria through multiple methods. Such a multilayer mode of action makes it far more difficult for bacteria to become resistant to the treatment. Moreover, the ability of nanomaterials to transport antibiotics to bacteria while also performing the function of a drug carrier leads to an increase in medication efficacy and a reduction in the amount of drug the body is exposed to. Drug resistance in biofilms is caused by numerous factors, including a reduction in drug incorporation through the extracellular matrix (ECM), a decrease in bacterial metabolic rates, a drop in drug concentration, and the transfer of resistant genes.^61^ Moreover, misuse and overuse of antibiotics are responsible for eighty percent of the multi-drug resistant or extensively drug-resistant microorganisms, and infections caused by these germs are linked with significant ill effects. Due to the proliferation of multidrug-resistant bacteria and other drug-resistant diseases, there are currently insufficient alternatives for treatment and prevention.^62^ Therefore, nanomaterials have the potential to meet the demand for alternate treatment alternatives for treating microbial infections, which has arisen because this need exists.

Nanotechnology is widely known as a potential therapeutic approach because of its high effectiveness and treatment response against germs. It provides a viable option for treating most bacterial illnesses, particularly those involving multi-drug-resistant microorganisms.^63^ Furthermore, nanomaterials that can kill microorganisms in response to various internal and external stimuli while providing improved drug delivery and release are intriguing techniques.^64^ Nonetheless, nanomaterials may be utilized independently or with antibiotics, resulting in strong synergistic results. Table 1 demonstrates how nanomaterials may work to prevent antibiotic resistance. Furthermore, the effectiveness of nanoparticles against bacteria resistant to many drugs, their modes of action and physical characteristics, and the benefits and drawbacks of using antimicrobial nanomaterials are outlined in Tables 2 and 3, respectively.

**Table 1:** Nanomaterials for the elimination of antibiotic resistance

Antibiotics		Mechanism of action	Possible approaches of nanomaterials to avoid resistance	Ref.
Class	Resistance mechanisms of choice			
Peptide antibiotics	Modifications to the outer membrane	Disruption of either the cell wall or the membrane	Physical damage to the cell membrane, which restricts the emergence of resistance; flexible design freedom and distinctive physio-chemical features, which may be exploited to optimize disruptive actions	65–70
β-Lactams	Porin alterations, drug-modifying enzymes, and binding site modifications			
Glycopeptides	Modifications to the binding site			
Quinolones	Modifications to the binding site and porins	Intracellular component damage	Penetration by membrane fusion overcomes resistance from restricted antimicrobial access, the capacity to inhibit efflux pumps and the availability of numerous active groups that target broad rather than specialized routes	60, 66, 68, 71 and 72
Aminoglycosides	Drug-modifying enzymes			
Macrolides	Binding site changes, efflux pumps			

**Table 2:** Nanomaterial efficacy against multidrug-resistant bacteria, as well as modes of action and physical properties

Nanomaterials	Size nm	Factors affecting antimicrobial activity/toxicity	Antibacterial mechanisms	Targeted bacteria and antibiotic resistance	References
Aluminum (Al)	10–100	Size, shape, and stability of the particles	Reactive oxidative stress leads to the breakdown of cell membranes	*E. coli*	73 and 74
Copper (Cu)	2–350	Concentration and size of the particles	DNA breakdown, loss of cell membrane potential, formation of reactive oxygen species (ROS), oxidation of proteins, and lipid peroxidation	*A. baumannii*, *E. coli*: multi-drug resistant (MDR)	74, 75, 76 and 77
Gold (Au)	1–100	Roughness and size of the particles	Rupture of the bacterial membrane, reduction in ATPase activity, reduction in the potential across the membrane, development of holes in the cell wall, disruption of the respiratory chain, and fall in tRNA binding to the ribosomal subunit	*Staphylococcus aureus*: Methicillin-resistant (MRSA)	76, 78 and 79
Iron oxide	1–100	It possesses a high level of chemical activity, has a propensity to form aggregates, and, when exposed to air, undergoes oxidation, which causes a reduction in magnetism and dispersibility	ROS-generated oxidative stress: singlet oxygen, hydrogen peroxide, hydroxyl radicals, & superoxide radicals	*E. coli*: MDR, *K. pneumoniae*, MRSA	76
Magnesium oxide (MgO)	15–100	Size, pH, and concentration of the particles	Alkaline effect, electrostatic interaction, ROS production, and lipid peroxidation	*E. coli*, *S. aureus*	73
Silver (Ag)	1–100	Size and shape of the particles	Disintegration of bacterial membranes, production of reactive oxygen species (ROS), suppression of cytochromes involved throughout the electron transport system, intercalation between DNA bases, suppression of cell wall formation, rise in the permeability of the membrane, adherence to cell surface resulting in damage to lipids and proteins, lysis because of the depletion of the proton gradient, ribosomal degradation	*Escherichia coli*: MDR, MRSA, *Klebsiella pneumoniae*, vancomycin-resistant *Enterococcus* (VRE), *Pseudomonas aeruginosa*, *A. baumannii*: carbapenem and polymyxin B-resistant, *Staphylococcus epidermidis*, *P. aeruginosa*: carbapenem- resistant, extended-spectrum beta-lactamase (ESBL)-producing organisms, carbapenem-resistant *Enterobacteriaceae* (CRE)	77, 79 and 80
Silica (Si)	20–400	Size, shape, and stability of the particles	Reactive oxidative stress leads to the breakdown of cell membranes	MRSA	78
Titanium dioxide (TiO_2_)	30–45	Structure, shape, and size of crystal	Cell surface adsorption and the production of ROS	*S. aureus*, *E. coli*, *Enterococcus faecium*, *P. aeruginosa*	73
Zinc oxide (ZnO)	10–100	Concentration and size of the particles	Production of reactive oxygen species, rupture of membranes, adsorption to cell surfaces, and damage to lipids and proteins	*E. coli*, ESBL-producing *E. coli*, MRSA, *Klebsiella oxytoca*, *Enterobacter aerogenes K. pneumoniae*	74, 81 and 82

**Table 3:** Antimicrobial nanomaterials' advantages and disadvantages.^83^

Advantages	Disadvantages
Reduced likelihood of developing resistance to bacteria	A high level of exposure throughout the body to medications that have been locally delivered, along with the appropriate dosages to achieve the desired therapeutic effect
Longer lifespan of therapeutic effects as a result of delayed elimination	Nanotoxicity affects the brain, kidneys, lungs, liver, metabolic pathways, germ cells, and other tissues and organs
A wide range of therapeutic effectiveness	Inability to characterize nanomaterials using methods that are not influenced by their physical characteristics
Minimal immunosuppression	
Chemical antimicrobials with fewer adverse effects	A high level of exposure throughout the body to medications that have been locally delivered, along with the appropriate dosages to achieve the desired therapeutic effect
May pass through tissue walls such as the blood–brain barrier	
Enhanced ease of dissolution	Accumulation of nanomaterials administered intravenously in the body's cells and organs
Delivery of drugs to specified areas using accumulation	
Release of the drug under control	

## 2. Synthesis of nanomaterials

**Fig. 4:**
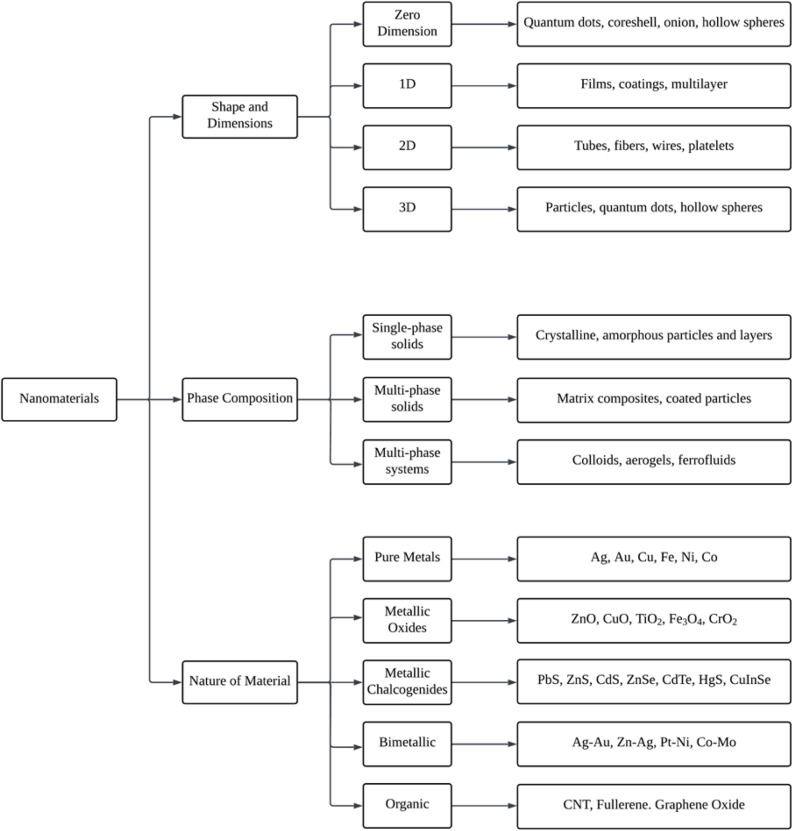
Representative schematic flowchart of the nanomaterial's classification (Reprinted from^84^ with permission from Elsevier).

There is a wide variety of nanoparticles that can be found widely in nature or that can be manufactured artificially. Even though natural nanoparticles are present in living organisms, it is assumed that they have been present in the biosphere since the Earth was first formed.^84^ As seen in Fig. 4, nanoparticles are separated into various categories according to their size and dimensions, phase composition, and the kind of material they are made of.

**Fig. 5:**
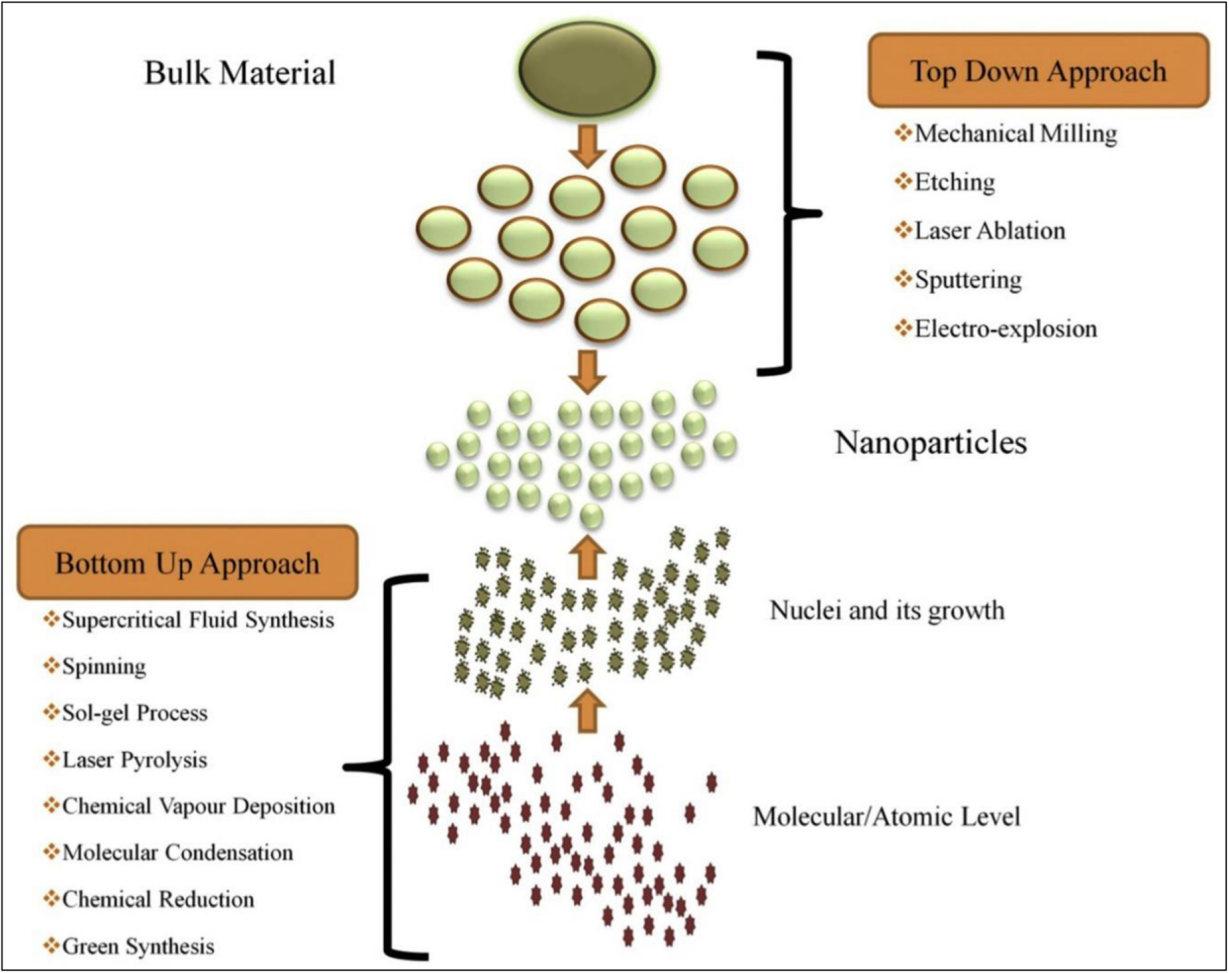
Schematic drawing related to the synthesis of nanomaterials (NPs) *via* top-down and bottom-up approaches (Reprinted from^84^ with permission from Elsevier).

Furthermore, top-down and bottom-up methods are the two primary strategies that can be utilized when fabricating nanomaterials. The top-down technique includes slicing massive amounts of material into smaller, self-assembled nanoscale units.^85^ On the other hand, the top-down method is turned on, resulting in the term “molecular nanotechnology,” which refers to assembling a specific structure by linking atoms, molecules, clusters, or clusters inside clusters, respectively, or by self-organization. Fig. 5 illustrates the synthesis of nanomaterials.

### 2.1. Top-down approach

The top-down approach is a complex synthesis technique used for nanoparticles. The transformation of materials in bulk into those on a nanometric scale underpins this approach.^86^ This method may break large quantities of material into much smaller nanoparticles. The manufacture of irregularly shaped and extraordinarily tiny particles is not a good fit for top-down procedures despite these methods being easy to employ.^87^ Nonetheless, the most significant drawback of using this strategy is its difficulty in producing particles of the appropriate size and form.

#### 2.1.1. Mechanical milling

Ball milling is the top-down approach's mechanical technique that is simplest and most efficient. When contrasted with alternative mechanical top-down techniques, the ball milling process is typically advantageous for synthesizing various nanoparticles. Fracture of particles, plastic deformation, and cold-welding are three significant aspects that impact the manufacturing process and assist in preserving the quality of nanomaterials.^88^

In addition, the ingredients are mixed in a sealed container throughout this procedure. Then, small stainless steel, ceramics, and glass rocks create shear force during grinding. After that, bulk items are placed in an air-tight container. Finally, the grinding process converts bulk materials to fine nanoparticles.^89,90^

#### 2.1.2. Laser ablation

The process of laser ablation, which uses a pulsed laser to remove molecules off the surface of a substrate to form micro and nanostructures, has a wide variety of applications in various materials.^91^ Laser ablation (LA) is a top-down technique that removes solid material from a substrate by putting the laser beam over the substrate. A popular LA method includes concentrating one laser beam beyond the metal object immersed in liquid. Then, the procedure generates vapor and molten metal or plasma droplets, which combine with a liquid medium to produce certain chemicals and grow as nanoparticles.^92^

Furthermore, adjusting the laser settings and liquid medium may modify the nanoparticles' shape, composition, and other features. Nonetheless, as a liquid, substances such as chloroform, acetone, water, isopropyl alcohol, and ethanol, amongst others, may be used, whereas metal is most often utilized as the solid target.^93^

#### 2.1.3. Sputtering

The production of nanomaterials may be accomplished by a technique known as sputtering, which involves hitting a solid plate with elements having higher energy, like gas or plasma. Sputtering is often regarded as an efficient production process for creating thin nanomaterial film.^94^ The length of the annealing period, temperature, materials used for nanoparticle fabrication, and other factors all have a role in determining the deposited layer thickness. In addition, nanoparticles' sizes and shapes are also determined by these characteristics, in addition to their shapes. Moreover, sputtering is a method in which nanomaterials are accumulated on the surface of a substrate by the ejection of particles due to the assault of high-energy ions.

For plasma-based sources to function, an electric potential must be applied between the electrodes when the environment is in the gas phase and has low pressure. Plasma is the name given to the ionized gas that results when accelerated electrons hit gas atoms; sputtering may be done using plasma ions, the most straightforward source of sputtering material.^95^

#### 2.1.4. Thermal evaporation

The thermal evaporation technique is considered endothermic, and the primary reaction during this process occurs when exposed to a hot temperature.^96^ The specific decomposition temperature is the temperature's value when reactant metal complexes break down. Furthermore, the decomposition process also involves several factors, including reaction time and pressure. The reactant being broken down creates nanoparticles that are stable and of a tiny size. Thermal evaporation is among the most often used ways to manufacture stable nanoparticles with the capacity for self-assembly. However, evaporation at high temperatures creates thin films on many substrates. This method is one of the several approaches used in manufacturing inorganic nanoparticles.^97^ In thermal evaporation, the heating of the target material is accomplished by an electrical current. In contrast, in electron-beam evaporation, the source material is heated by being bombarded by an electron beam.

### 2.2. Bottom-up approach

The production of nanocrystals from particles in the atomic range is the premise behind this procedure, which is another reason why it is regarded as a valuable technique. The top-down method's antithesis, the bottom-up approach, is the exact opposite of it. Through the top-down approach, the proliferation and self-assembly of molecules and atoms may result in the formation of nanomaterials with a chemical composition, structure, and size that can be precisely specified.^87^

#### 2.2.1. Chemical vapor deposition (CVD)

When heated, bombarded by photons, or exposed to a plasma, chemical vapor deposition (CVD) occurs. This may include the dispersion of any gaseous chemical or the chemical interactions between gaseous reactants.^98,99^ The conventional plasma-assisted chemical vapor deposition technique. The following concisely summarizes the primary stages involved in an average CVD process.

1. Transfer of gaseous species that have been involved in a reaction to the substrate surface,

2. Species adsorption onto that surface,

3. The substrate's surface catalyzes a heterogeneous reaction on the surface,

4. The movement of the species from the surface to the growing locations,

5. Film nucleation and growth upon that substrate,

6. Desorption and transfer of gaseous reaction products.

#### 2.2.2. Physical vapor deposition (PVD)

In this technique, the target functions as a cathode and is equipped with an evaporation source that vaporizes the material from a source that may be either liquid or solid. The particles of atomic size are evaporated during the heating process with an electron beam, and the collision of the newly freed particles with the gas molecules that have been injected into the chamber causes the particles to accelerate. This results in the generation of plasma, which travels *via* a deposition compartment and then a vacuum pump before reaching the surface, where it condenses and causes the development of a thin coating. In addition, these films might have a thickness that ranges from a few nanometers to thousands of nanometers, depending on the circumstances.^100,101^

#### 2.2.3. Chemical reduction

**Fig. 6:**
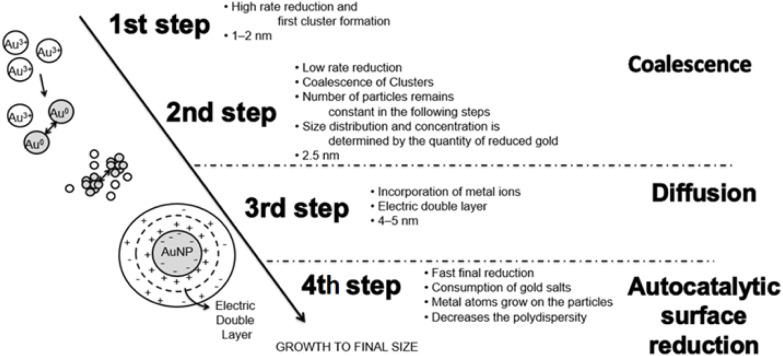
Stages involved in producing gold nanoparticles by chemical reduction (Reprinted from ref. 103 with permission from Elsevier).

The chemical reduction process is the most flexible method for manufacturing nanomaterials since it is so easy to use and only requires a relatively simple apparatus. Steps involved in producing gold nanoparticles using chemical reduction process are illustrated in Fig. 6. The Brust governs most chemical reduction techniques–Schiffrin two-phase process, in which chemical reduction occurs at the oil–water interface. The thiolated atoms are adsorbable in the organic phase and then stabilize in the subsequent step, which follows directly on the heels of the first step.^102^

#### 2.2.4. Hydrothermal approach

Hydrothermal technique is one of the most popular nanoparticle production.^104^ Crystallization is a process that may occur directly from solutions during the process of material synthesis *via* the use of hydrothermal or solvothermal processes. This process will typically entail two parts: nucleation or crystal formation and consequent development. The phenomena that lie behind regulating size and morphology *via* modifying the processing parameters are the cumulative nucleation and growth rates depending on the level of supersaturation.^105^ Nonetheless, since water is utilized as a solvent in the hydrothermal process, this procedure is referred to as the hydrothermal process. The procedure is carried out within an autoclave, a kind of pressure vessel made of steel, and here, the processing conditions may be controlled by changing the temperature and/or the pressure. Furthermore, the temperature is raised above the point at which the water would boil, achieving the desired level of vapor saturation.

#### 2.2.5. Sol–gel approach

The sol–gel method is widely regarded as a unique synthetic method for fabricating high-quality nanomaterials like metal oxide nanomaterials and composites of mixed oxide.^106^ In addition, over the last several decades, the automotive industry has made significant investments in the research and development of nanocomposites that are disseminated inside polymer matrices to create lightweight components.

The morphology and physical features of the materials may be precisely controlled using this process, and the sol–gel method can, in general, be broken down into five primary stages, which have been briefly explained later in this section.

✓ Hydrolysis: Oxygen (O_2_) is required to form a metal oxide that may be provided when creating metal oxide nanomaterials utilizing either aqueous or organic solvents. The technique is called the aqueous sol–gel approach, while water is the reaction media. On the other hand, when an organic solvent is utilized in the process as the reaction medium, the method is referred to as the nonaqueous sol–gel. However, using either a base or an acid is helpful to hydrolyse the precursors. The following is a detailed description of the typical chemical reaction during hydrolysis.M − OR + H_2_O → MOH + ROH (hydrolysis)Here, M and R have expressed metal and alkyl groups, respectively. In addition, the alkyl group is denoted by C_*n*_H_2*n*+1_

✓ Polycondensation: In this stage, neighboring molecules condense whilst the material is still liquid. This results in the elimination of alcohol and water, the development of metal oxide interconnections, and the growth of polymer films to microscopic dimensions. The basic chemical reaction which takes place during the condensation process is specified below,M − OH + XO − M → M − O − M + XOH (condensation)Here, metal and alkyl groups have been expressed by M and X, respectively. In addition, the alkyl group is denoted by C_*n*_H_2*n*+1_

✓ Aging: The aging process causes ongoing shifts and modifications to the structure and characteristics of the gel. Porosity goes down, and the space between colloidal particles goes up as a result.

✓ Drying: Drying is more difficult since organic constituents and water are separated to yield gel, affecting the substance's structure. Many drying methods include freeze-drying, atmospheric/thermal drying, and supercritical drying. Each method influences the development of the gel network in a distinct way from the others.

✓ Thermal decomposition: In the final step, a thermal treatment known as calcination is carried out to remove residues as well as molecules of water from the intended sample. The temperature at which the calcination is carried out is a significant factor in influencing the size of pores and material density. Consequently, calcination is carried out to yield nanoparticles.

#### 2.2.6. Green synthesis

A growing amount of research is being conducted on synthesizing nanoscale metals using physical, chemical, physical, and green synthesis approaches.^107–110^ Nevertheless, due to problems with hazardous chemical release, high energy consumption, usage of complicated equipment, and synthesis conditions, physical and chemical procedures are progressively substituted by green synthesis approaches.^111^ The following may be a concise summary of the methodologies^112^ and processes for the green or environmentally friendly synthesis of various nanomaterials.

To get the desired nanoparticle, first acquire plant extract, combine the extract with a metal salt solution under a predetermined set of circumstances, decrease the size of the metal particles, and finally carry out filtering and any other necessary operations.

## 3. Physicochemical features of the nanomaterials

Antibiotics made of chemicals can be quite hazardous to their users as the composition of bulk materials primarily influences their toxicity. However, the physicochemical properties of nanomaterials, namely size, shape, surface area, composition, *etc.*, significantly impact their toxicity.^113^ In addition, using phytochemicals in synthesizing biocompatible NPs in various concentrations makes the NP non-toxic, making it ideal for antibiotic application. Some important physicochemical properties of the nanomaterials are specified in the following subsections.

### 3.1. Size

Surface area and particle size are important factors in how materials interact with biological systems. Surface area increases exponentially in relation to volume with the decrease of the material size, increasing the surface reactivity of the nanomaterial both on its own and in its surrounding environment. Notably, how the system reacts, distributes, and gets rid of the materials depends on the size of the particle and surface area.^114^ The size of the material has been found to affect a number of biological methods, including cellular uptake, endocytosis, and the endocytic pathway's particle processing effectiveness.^115,116^ The capacity of nanoparticles to penetrate biological systems and alter the structure of different macromolecules, so interfering with vital biological activities, is often responsible for their size-dependent toxicity.^117,118^

The size of nanoparticles also affects how they behave pharmacologically. NPs smaller than 50 nm, when administered intravenously, transverse almost all tissues quickly and can cause potentially toxic manifestations in different tissues; however, NPs larger than 50 nm, especially positively charged particles between 100 and 200 nm, are easily absorbed by RES and do not travel to other tissues. While RES protects other tissues through clearance, RES organs, including the spleen and liver, are the primary oxidative stress targets.^119^

Numerous toxicological investigations have indicated that, in comparison to bigger particles of the same substance, smaller nanoparticles with diameters less than 100 nm have detrimental effects on respiratory health.^120,121^ The human respiratory system displays varying fractional depositions of inhaled particles based on size. It has been noted that all locations are deposited with ultrafine particles with diameters less than 100 nm. In contrast, the tracheobronchial region is deposited with particles less than 10 nm, and the alveolar region is deposited with particles between 10 and 20 nm.^122^ Consequently, it has been discovered that the translocation or distribution of NPs is size-dependent, which determines their toxicological concerns.

Furthermore, the level of oral toxicity typically increases as the size of a nanoparticle decreases, which in turn effects its toxicity when swallowed. One study found that the toxicity of copper nanoparticles in the mouth rose as their size decreased. More significantly, bigger particles remained benign even at increasing dosages, whereas smaller particles were somewhat hazardous.^123^

In general, biodegradable nanoparticles are probably less dangerous than nonbiodegradable ones. Semete *et al.*^124^ examined the biodistribution and *in vivo* toxicity of 200–300 nm-sized (poly (d, l-lactic-*co*-glycolic acid)) or PLGA nanoparticles. Following a seven-day oral dosing in mice, around 40% of the PLGA particles were detected in the liver, and the remaining particles were found in the kidney and brain with no discernible damage. Particle size can impact the breakdown of the polymer matrix in addition to its size-dependent toxicity because of its capacity to produce reactive oxygen species. It is not that fewer NPs were entering the brain in this instance; rather, the surface area to volume ratio increases significantly as particle size decreases, making it simpler for the polymer breakdown products to penetrate and be released.^125^

### 3.2. Shape

At the nanoscale level, a nanomaterial's shape or morphology is just as significant as its size. Nonetheless, the biological or toxicological link connected to this characteristic alone is the subject of very few studies. Nevertheless, metallic nanomaterials' shapes significantly impact a range of properties, such as electromagnetic, optical, and catalytic properties.^126–129^ As a result, it is thought that nanomaterial shape also significantly impacts nano-bio interfaces in addition to nanoparticle size. Apart from being the primary factor in cellular absorption, a material's size is crucial in determining its surface area at a given mass dosage. The form of the nanoparticles will generally contribute significantly to the total surface area. For example, a nanomaterial with an octagonal form will have a different surface area than a sphere of the same size. The greater catalytic activity of a nanomaterial with a bigger surface area increases its reactivity because surface atoms tend to retain unfulfilled high-energy bonds.^130^ Therefore, these nanomaterials will have a higher chance of interacting with cell biomolecules after successfully entering the cellular environment than their micron-sized counterparts, leading to direct cellular damage and the promotion of oxidative stress^131,132^

Nanoscaled fibers (like carbon nanotubes) are known to provide a significant risk of lung inflammation, much like other well-known inhalable fibers (like asbestos).^133^ Additionally, repeated exposure has been linked to a number of malignancies.^134–136^ Determining whether a single nanotube or a group of them has a specific hazardous impact is challenging. According to certain research, carbon nanotubes are more hazardous than silica dust or other ultra-fine carbon black. The majority of workers who were exposed to single-walled carbon nanotubes (SWCNTs) more than the permissible exposure limit (PEL) at the time had lung injuries.^137^ Interestingly, studies have demonstrated that carbon nanotubes (CNTs) kill specific kidney cells by preventing cell development due to a loss in cell adhesiveness.^138^ In addition to killing fish brains and causing water flea deaths, fullerene has been linked to serious lung damage in humans.^139–142^

### 3.3. Composition and crystalline structure

Research showing similar toxicities for various nanoparticle chemistries with identical dimensions cannot be ignored despite particle size being a major factor in determining a nanoparticle's toxicity. These studies that a nanoparticle's crystalline shape and content impact its toxicity concerns. Griffitt *et al.*^143^ found that TiO_2_ of the same dimensions did not cause any toxicity issues, while nano silver and nano-copper in their soluble forms caused toxicity in all tested organisms. This highlights the role of compositions in determining the toxicities of NPs. The study used algal species, daphnids, and zebrafish as models of various trophic levels.^143^

Their crystal structure can also influence the toxicity of nanoparticles. In the absence of light, rutile TiO_2_ NPs have been shown to cause lipid peroxidation, oxidative DNA damage, and micronuclei formation, while anatase nanoparticles with the same size and chemical makeup did not.^121^ Furthermore, upon interacting with water or another dispersion media, NPs can alter their structural structure. According to a report, ZnS nanoparticles reorganize their crystal structure in water, approaching the structure of a bulk ZnS piece.^144^

### 3.4. Surface area

The surface area is a crucial factor in demonstrating toxic manifestations (epithelial-induced inflammatory responses in the lungs and other organs) in rodents, according to a number of studies employing nanoparticles of various classes.^145^ An increase in the surface area of nanoparticles results in a dose-dependent enhancement of their oxidation and DNA-damaging capabilities, which are significantly greater than those of larger particles containing the same mass dose.^120,146^

Investigations on the detrimental effects of a large surface area-induced change in the band gap, decrease in melting point, and increase in reactivity revealed that these characteristics led to pulmonary inflammation, cytotoxicity, and *in vivo* toxicity.^147–149^ When utilized as drug carriers, additives, or cosmetics, a greater surface area may lead to increased reactivity with neighboring particulates, which may have adverse effects.^115^ The conclusion that can be drawn is that a significant increase in biological activity results from a reduction in particle size. As a consequence of reduced volume, a greater quantity of particles can be accommodated per unit area, leading to heightened pathophysiological toxicity mechanisms such as oxidative stress, reactive oxygen species (ROS) production, mitochondrial disruption, and others.^150^ The characteristics of nanoparticles that induce such pronounced biological toxicity remain to be ascertained. It is hypothesized that the total number of nanoparticles per unit volume may be significant rather than the size of the particles alone, which could account for toxicity. To comprehensively understand the correlation between the surface area of a nanoparticle and its biological toxicity, a cohort of scientists examined acute lung inflammation in the presence and absence of specific reactivity of various nanoparticle surface areas.^151^ No statistically significant variations in toxicity were observed according to size. However, pulmonary inflammation was significantly influenced by the total surface area. It has been unequivocally established that ultrafine and fine materials with substantial surface areas can induce pulmonary toxicity, as determined by testing the effects of treatment with titanium dioxide, carbon black, and various other particles.^152,153^ Cytotoxicity may also be significantly influenced by particle surface reactivity, defined as the ease with which individual particles aggregate. Additionally, dosing should be determined by the surface area of the nanoparticles.^154–156^

### 3.5. Surface charge

In addition to the dimensions and morphology of the nanoparticles, their antibacterial efficacy and cytocompatibility are significantly influenced by their surface charge.^157^ Positive charge nanoparticles are encouraged to adhere to bacterial internal components, including DNA, which possess negative charge in addition to their negatively charged surfaces. The binding efficiency of positively charged nanoparticles to bacterial cell surfaces is enhanced compared to negatively charged nanoparticles, owing to an electrostatic phenomenon.^158^ How nanoparticle surfaces form *in situ* complexes with bacterial cell surfaces remains a matter of debate, which impedes the development of a comprehensive comprehension of the relationship between binding and charge efficacy.^159^ The process of ion exchange facilitated by cationic nanoparticle deposition at sites of infection may enable access to bacterial cells.^160^ Several studies have demonstrated that cationic liposomes encapsulate antibiotics more effectively, facilitating the internalization of the drugs to the biofilm at reduced concentrations.^161^ On the other hand, some studies found that charge-negative nanoparticles could remarkably bind to bacteria, indicating that electrostatic repulsion is not the dominant force at work when it comes to nanoparticles' high surface energy.^162^ Mixed-charged nanoparticles, which disrupt bacterial cell walls *via* electrostatic and noncovalent attachment, have recently garnered considerable attention.^163^

### 3.6. Concentration

Concentration is a crucial last factor to consider while assessing nanomaterials' toxicity. According to research by Santos *et al.*,^164^ thermally carbonized and hydrocarbonized porous silicon particles were harmful to cells at concentrations of [mt]2 mg mL^−1^; for porous silicon particulates that are thermally oxidized, the non-toxic threshold value was 4 mg mL^−1^. Research on the toxicity of several nanoparticle types (TiO_2_, MoO_3_, Al, and Fe_3_O_4_) *in vitro* was done by Hussain *et al.*^165^ in BRL 3A rat liver cells. They c that mitochondrial activity will drastically deteriorate when subjected to Ag nanoparticles at concentrations of 5–50 μg mL^−1^. When Ag nanoparticle concentrations reached 100–250 μg mL^−1^, cell LDH leakage increased. Similar findings were made by Usenko *et al.*,^166^ who used embryonic zebrafish to test the toxicity of carbon fullerene and found that exposure to 200 μg mL^−1^ C60 and C70 significantly increased the number of abnormalities.

Thus, it is evident that nanomaterials' toxicity depends on their physicochemical properties. The comparison of the toxicity between traditional antibiotics and nanomaterials is specified in Table 4.

**Table 4:** Toxicity analysis of traditional antibiotics and nanomaterials.^167,168^

Aspect	Traditional antibiotics	Nanomaterials
Nature of toxicity	Specific drug-related reactions (*e.g.*, phototoxicity, ototoxicity, nephrotoxicity, *etc.*)	Diverse effects are influenced by size, shape, composition, *etc.*, and are often related to oxidative stress, DNA damage, and cell death
Mechanisms of toxicity	Primarily target bacterial structures or functions, affecting specific microbial processes	Act on diverse cellular targets, including mammalian cells. Nanoparticles can induce oxidative stress and damage various cellular components, leading to cytotoxicity
Long-term effects	Long-term effects may include antibiotic resistance and potential side effects in individuals	Long-term exposure, especially through incorporation into products like dental materials, raises concerns about systemic effects due to nanoparticle release

**Fig. 7:**
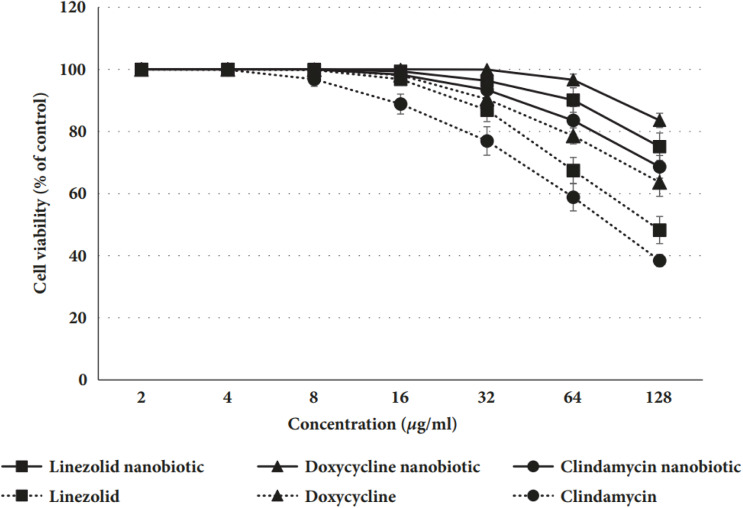
Comparison between some nanobiotics and traditional antibiotics.^169^

Mohamed *et al.*^169^ conducted an experiment on rats to determine the level of toxicity between nanobiotics and conventional antibiotics. After 24 hours exposure to various concentrations of linezolid, doxycycline, and clindamycin nanobiotics, the percentage of viable rat hepatocytes relative to the control group in comparison to their conventional antibiotic counterparts is depicted in Fig. 7. The values presented are the means (± standard deviation) of three separate experiments.

The results of the cell viability experiment, as shown in Fig. 7, confirmed that hepatocytes treated with nanobiotic formulations had greater percentages of live cells than those treated with traditional antibiotics, indicating that nanobiotics are more cytocompatible and less toxic.

## 4. Interaction of nanomaterials with microorganisms and antibacterial potential

Several mechanisms determine interactions between nanomaterials and bacteria, including electrostatic attraction, hydrophobic contact, the interaction between receptors and ligands, and van der Waals forces. Studying fundamental interactions between nanoparticles and bacteria gives vital knowledge for formulating innovative antimicrobial drugs. Nanoparticles have six primary antibacterial actions, which are as follows:^170–172^

(i) Direct contact with the cell wall and membrane of the bacterial organism,

(ii) Cell membrane penetration,

(iii) Production of reactive oxygen species (ROS),

(iv) Protein synthesis inhibition and damage of DNA,

(v) Damage to the pathway of metabolism and

(vi) Inhibition of biofilm formation.

**Fig. 8:**
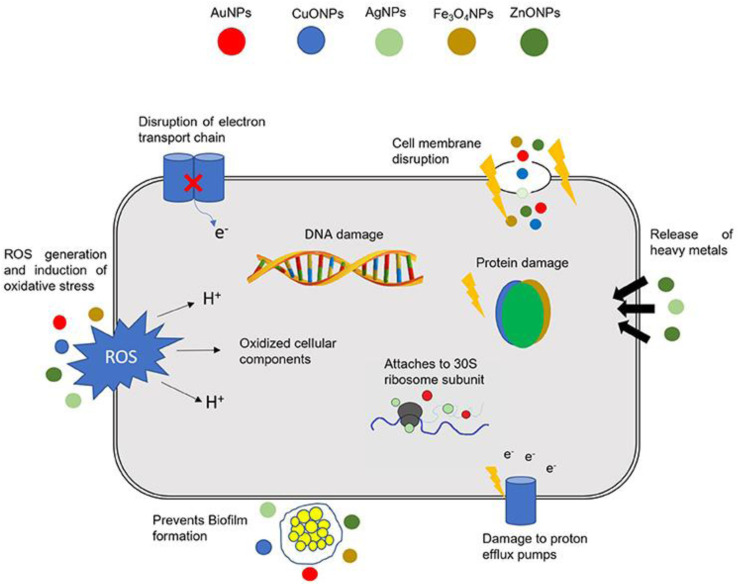
Nanomaterial action mechanisms in bacterial cells (Reprinted with permission from ref. 174, An open access article distributed under the terms of the Creative Commons Attribution License).

Fig. 8 demonstrates the mechanism of nanomaterial antibacterial action mentioned above. In addition, nanomaterials can potentially be of tremendous value in the fight against MDR bacteria as they do not exhibit the same modes of action as conventional antibiotics.^173^

As can be seen in Fig. 8, bacteria have developed several defense mechanisms that allow them to avoid being killed by antibiotics. This is how bacteria have survived the widespread use of antibiotics. Biofilm formation independently confers antibiotic resistance, which is a distinct phenomenon from the genetic acquisition of resistance.^175^ Bacteria can self-colonize, which results in the formation of biofilms. Infections caused by biofilms are difficult to cure because the extracellular matrix formed by the bacteria forms a microenvironment inside the host. This makes it possible for bacteria to elude the immune system's defenses and dramatically increases their resistance to conventional antibiotic treatments.^176^ The creation of biofilm therapies relies heavily on the nanomaterials' ability to penetrate biofilms and deposit them inside. Nanomaterials are uncharted territory for bacteria; they can sidestep resistance mechanisms posed by conventional antibiotics and target biofilms produced by bacteria.

**Fig. 9:**
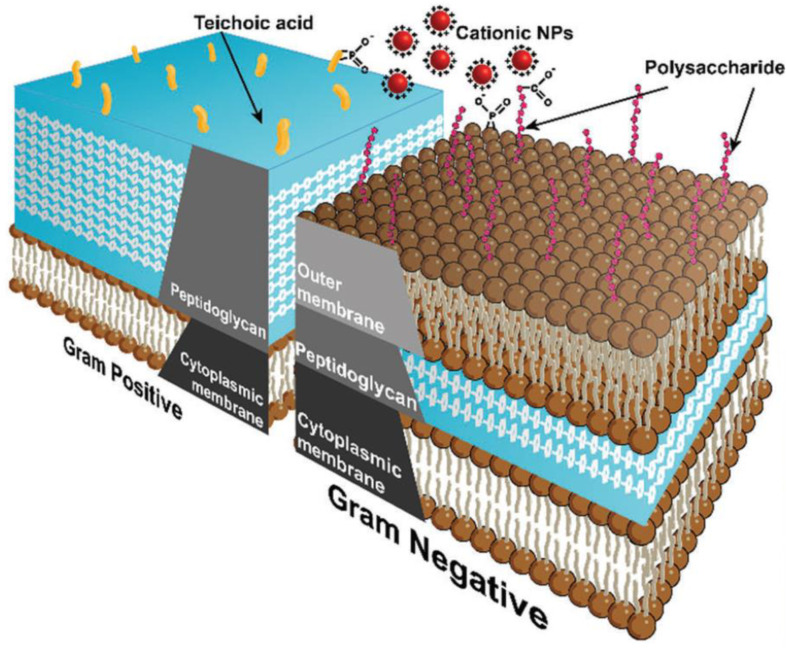
Schematic diagram showing cell wall structures of Gram-positive and Gram-negative bacteria.^13^

Antimicrobials can't penetrate the bacterial cell membrane since they have been modified through time to behave as a physical barrier. The bacterial cell wall composition is the primary factor determining whether bacteria are categorized as Gram-negative or Gram-positive. Teichoic acids are found in the cell walls of Gram-positive bacteria, whereas lipopolysaccharide is in the outer membranes of Gram-negative bacteria. Both types of bacteria include phosphate groups, which cause the surfaces of the bacteria to be negatively charged. This extremely polar environment restricts hydrophobic antimicrobials' membrane penetration and bacterial action. Gram-negative bacteria are distinguished by the presence of an additional layer that may be found between the plasma membrane and the outermost layer. However, this layer is far thinner compared to the outer layer of Gram-positive bacteria. As a result, it is more vulnerable to penetration by antimicrobial nanomaterials, which may eventually lead to the cell's demise^177,178^ (Fig. 9).

The fundamental method *via* which nanoparticles might harm bacterium is oxidative stress induced by reactive oxygen species (ROS) since bacteria can keep a balance throughout the formation of ROS under normal circumstances. Nonetheless, this equilibrium is influenced when it touches some nanoparticles. This results in excessive ROS, eventually changing the state of oxide-reduction molecules, favoring the oxidation of biological constituents. Once the nanoparticles have broken through the cell wall, they tend to release ions and produce ROS *via* a process known as adsorption, which is a kind of diffusion.^179^ Moreover, DNA damage and impairment of protein synthesis are other processes linked to metal nanoparticles in most studies. These typically induce a breakdown in enzymes, ribosomal component proteins, and other proteins generated in bacterial cell membranes, as well as compaction, degradation, and bacterial DNA fragmentation, reducing the physiological function of genes.^180^ However, it has been revealed that when nanostructures enter the bacterial cell, there are alterations in the metabolism of the bacterium, which in turn causes cell damage to the membrane, which in turn induces oxidative stress, which ultimately leads to the killing of the bacterium.^181^ Finally, nanoparticles interacting with bacterial biofilms connect with EPS, allowing any chemical molecule to reach the bacterium and destroy the cell.^38^

In contrast to antibiotics, nanomaterials may have more contact with cells owing to a larger volume-to-surface ratio, making nanoparticles adaptable for use as strategic active ingredients. Nanomaterials on their own often contain complicated processes that function concurrently to prevent the generation of genetic alterations in bacteria and to restrict their development.^182^ In addition, nanoparticles as antibiotic adjuvants also work as protectors, meaning they may boost serum concentrations of the medications and protect against the enzyme system of the target. On the contrary, antibiotics lose some of their effectiveness over time, and the body of a human can only absorb half of the medication before the other half is flushed out during urination, reducing the antibiotic's effectiveness. Techniques for interacting nanoparticles with microorganisms are outlined in Table 5. Further, the investigations listed in Table 6, which various researchers carried out, showed that using metal nanoparticles functionalized with antibiotics resulted in a more potent suppression of multi-resistant bacteria, demonstrating the antibacterial potential of several nanomaterials.

**Table 5:** Techniques of interacting nanoparticles with microorganisms

Nanomaterial	Methods of interacting nanomaterials with microorganisms	References
Au	Strong attraction due to electrostatic force, interactions with the membranes of the cells	183
Ag	Ionization with Ag^+^ being released, damage of DNA, interference with the cell membrane and the electron transport system	184
CNTs	Oxidation of the proteins and lipids that are found in cell membranes, ROS causes cell membrane deterioration	185
Chitosan	Inhibition of enzyme activity, chelation of several metals, including trace elements, rupture of the membrane, and an increase in its permeability	186
NO-releasing nanoparticles	The release of NO and reactive oxygen species (ROS) generation	187
ZnO	The formation of H_2_O_2_, the aggregation of nanoparticles inside of cells, disruption to the cell membrane, ionization with Zn^2+^ being released	188
Fullerenes	Boosting the activity of invading neutrophils, loss of structural integrity of the cell membrane	139
TiO_2_	Deterioration of the cell membrane and walls, reactive oxygen species (ROS) generation	189
Nanoemulsion	Disruption of the membrane as well as spore coat	190

**Table 6:** Resistance against microbial pathogens using nanomaterials

Nanomaterials	Target microorganisms	Size	Mechanism of activity	References
Ag	*S. typhimurium*	35 ± 15 nm (spheroide)	The combination of Ag nanoparticles and the antibiotic caused damage to the membrane	191
	*B. subtilis*			
	*E. coli*			
	*S. aureus*			
	*S. haemolyticus*	8–21 nm (spherical)	ROS production and augmentation	192
	*S. epidermis*			
	*P. aeruginosa*	10 nm	Nanoparticles of silver may infiltrate cells, blocking enzyme systems in the respiratory chain and affecting DNA synthesis	193
	*E. coli*	26 nm (spheres)	Antibiotic mode of action and ROS production	194
	*A. actinomycetemcomit-ans*			
	*S. aureus* MRSA			
	*P. aeruginosa*			
Mesoporous silica	*A. baumannii*	50–100 nm (spherical)	Mechanisms of antibiotic action	195
Au	*S. aureus* MRSA	8 ± 2 nm	ROS production resulting from the impact of the antibiotic	196
	*S. aureus*	33 ± 14 nm	The interaction between bacteria and antibiotics results in an increase in the concentration of the antibiotic due to the combination of antibiotics and NPs. Moreover, the multivalent appearance of amoxicillin prevents the microbial efflux pump from functioning	197
	*E. coli*			
	*S. epidermis*			
	*S. aureus*	25 nm	The capacity of Au nanoparticles to interact with bacterial cell walls and rupture them and their potential to impair bacterial metabolism by interfering with bacterial DNA makes Au nanoparticles a potent bactericidal agent	182
	*P. aeruginosa*			
	*E. coli*	5 nm	—	198
	*A. baumannii*	200 nm	The mechanism of action of antibiotics is enhanced due to the NPs	199
	*Klebsiella pneumaniae*	35 nm		
	*P aeruginosa*	30 ± 20 nm (irregular)	Biofilm harm	200
TiO_2_	*P. aeruginosa*	64 ± 0.14 nm (irregular Spheres)	When combined with the nanostructure, the antibacterial agent boosts the synergistic action of the antibiotic, which may impede cell growth	201
	MDR *S. Aureus*	20 nm	ROS destroys cell components	202
	*S. aureus*			
	*E. coli*			
	*B. subtilis*			
	*S. typhimurium*			
	*K. pneumoniae*			
Compounds of ferrocene and carborane containing nano-sized TiO_2_	MDR *A. baumannii*	20–95		203
Magnetic nanoparticles with Fe_3_O_4_ at their TiO_2_ core or shell	*S. aureus* MRSA	20–100 (NO as donor particle)		204
	Multiantibiotic-resistant *S. pyogenes*			
Iron oxide	*Staphylococcus aureus*	9 ± 4 nm	ROS produces oxidative stress, which is harmful to proteins and DNA and causes damage to both	205
Zinc oxide	*K. pneumoniae and E. coli* with a broad range of β-lactase production	12–60	Within the bacterial cell, the production of oxidative stress	206
	*S. epidermidis* MRSE			
	*S. aureus* MRSA		Internalization of nanoparticles inside cells leads to damage and disorganization of the cell membrane, increasing the membrane's permeability	206
	*Streptococcus agalactiae* MRSA			
Nitric oxide	*S. aureus* MRSA	20–100 (NO as donor particle)	When reactive nitrogen oxide species, also known as RNOS, attach to heme proteins, they do so in an irreversible manner, ultimately resulting in heme loss from the protein	207 and 208
	*Acinetobacter baumannii*			
	*E. coli*		Liposomes' lipids were peroxidized by the RNOS that was generated	209
	*P. aeruginosa*			
	*E. faecalis*			
	*K. pneumoniae*			
	*S. pyogenes*			
Copper–cotton nanocomposites	*A.baumannii*	5 nm	Copper nanoparticles have a biocidal effect because, when they come into contact with water, Cu nanoparticles disperse their bound Cu(ii) ions into the surrounding environment, creating an environment that is hostile to microorganisms. These copper ions form covalent bonds with the SH and COOH groups found on the protein molecules that make up the cell wall of bacteria	210
Chitosan/silver nanocomposite	*S. aureus* MRSA	43–55 nm	Chitosan interacts with ionic groups on the surface of cells to improve permeability or chelates trace elements needed for enzyme function. Silver nanoparticles' antibacterial activity is connected to their ions	211
Chitosan–nanosilver composite				
ZnTe/dendrimer nanocomposites	*E. coli* MDR	3 nm	Amine-ended ZnTe/dendrimer nanoparticles were charged positively and preferred negatively charged bacterial membranes. This gave them great penetrating power, making them efficient in binding to substrates on outer membranes and cell membranes	212
	*V. cholerae* MDR			
TiO_2_/Ag nanocomposite	*A. actinomycetemcomitans*	20–50 nm	TiO_2_/Ag nanocomposite can inhibit the growth of bacteria because of the electrostatic repulsion between *E. coli*, which has a negative charge, and the nanocomposite, which has a high positive charge, stimulates the contact between bacterial cells and silver nanoparticles	213
	*S. mutans*			

## 5. Application of nanomaterials in medical science

Nanotechnology is extensively employed in contemporary biomedicine, particularly in the field of drug delivery. A significant obstacle in attaining the intended bioavailability and suitable levels of drug efficacy is the considerable difficulty posed by the near-insolubility of an estimated 60% of all drug entities in development.^214^ The advancement of nanotechnology has facilitated not only the encapsulation of drugs to increase their solubility but also their permeation through membranes, which results in extended circulation durations and improved overall efficiency. Targeted therapeutics have emerged with expanding knowledge in this domain and implementing a multidisciplinary strategy. These enable the desired drug to selectively target a specific site of action within the body. As a result, numerous toxic, immobile, and impermeable substances have been permitted to progress to clinical trials as prospective treatments.^215^

Nanomaterials are used in medical diagnoses like imaging diagnosis, laboratory diagnosis, genetic disease diagnosis, and tumor early diagnosis. They are also used in the synthesis of medical instruments—for instance, nanoprobes, nanorobots, handheld disease diagnosis instruments, and nanosensors. Most importantly, nanomaterials are becoming very common in tissue engineering to fabricate nanobones. Nanomaterials are also utilized as nanoscale red blood cell substitutes. In the drug industry, applications of nanomaterials in therapeutic drugs include antibacterial nano-drugs, antiviral nano-drugs, antitumor nano-drugs, analgesic and anti-inflammatory nano-drugs, encapsulating hormone drugs, nanometer polypeptide, and protein drugs. Furthermore, nanomaterials are very efficient and widely used in nanogene medicine, gene therapy of cancer, nano vaccines, and as radiosensitizers in radiation therapy.^216^

Nanomaterials are being extensively studied in medical science for drug delivery due to the mutual penetration of nanoscience and current technology.^217^ Drug delivery aims to maximize bioavailability at certain sites in the body and over an extended period. Molecular targeting using nanoengineered carriers may be one way to do this.^218^ The following features of medications transported by nanocarriers are different from those of conventional pharmaceuticals.

⁃ Nanoscale carriers can encapsulate hydrophobic drugs due to their substantial surface area. This results in enhanced drug solubility and diminished reliance on co-solvents, customary in conventional drug formulations, to mitigate adverse effects.^219^

⁃ Through the endothelial cell gap, nano-drug carriers can traverse into the lesions, in addition to traversing the blood circulation into the capillaries. Phosphocytosis enables the cellular uptake of drugs transported by nanodrug carriers. Significant improvements will be observed in the bioavailability of the drugs.^220^

⁃ Tailored nanodrug carriers, including magnetic NPs and drug-loading NPs modified with folic acid, facilitate the efficient delivery of drugs to the intended tissue. This reduces the required dosage for drug administration and mitigates the associated adverse effects.^221^

⁃ When utilizing nanocarriers to deliver drugs, as opposed to simply administering them, it is possible to attain targeted drug therapy that is highly bioavailable while simultaneously mitigating drug adverse effects, which results in reduced treatment costs and dosage.^222–224^

⁃ Nanomaterial drug carriers can reduce the frequency of drug administration by enhancing the duration and efficacy of drug concentration in the blood and extending the half-life of drug elimination.^225^

⁃ Nanomaterial drugs have the capability to traverse various cellular biological membrane barriers, including those between the blood and eyes, brain, and blood vessels. This capability enables the drugs to target the lesions effectively.^226–228^

## 6. Advantages of NMs based antimicrobial over chemical antibiotics

Antibiotics are substances that, when administered correctly, inhibit the development of pathogens, combat certain diseases, and potentially preserve lives. These are the fundamental pharmacological options for biofilm and planktonic infections.^229^ On the other hand, Nano antibiotics (nAbts) refer to antibiotic molecules in their purified form that have undergone synthetic growth to achieve a minimum average diameter of 100 nm in a single dimension, or engineered nanoparticles (NPs) that contain the antibiotic molecules.

Conventional or chemical antibiotics often induce bacterial mortality through various mechanisms, including inhibition of protein synthesis, disruption of cell wall structural components, and inhibition of proton transport across the cell membrane.^230^ In response to stimuli, multifunctional antimicrobials encapsulated in NPs interact with the bacterial cell wall surfaces and membranes, thereby enhancing drug distribution to target sites and passage through membranes. Moreover, nAbts possess a controlled, target-specific release mechanism that enables their administration in a single dose in contrast to the majority of conventional antibiotics, which necessitate the administration of numerous dosages for systematic release.^231^ Likewise, when comparing chemical or traditional antibiotics to nanoparticle-based delivery systems, the dosage of antibiotics administered through such systems offers several additional advantages. These include reduced dosage and frequency requirements, sustained drug release that enhances targeted drug delivery, and drug efficiency to a particular site within the bacterial cell.^231,232^

Unlike molecular substances, nanoparticles (NPs) possess a highly capacious contact area due to their distinctive surface area and high surface-to-volume ratio. Antibiotics are delivered to the intended sites of action, while the NPs activate an array of antibacterial defenses concurrently when they are combined with antibiotics. For the treatment of multi-drug resistant (MDR) strains, NPs, therefore, offer a greater number of functionalities than the effects of a single medication or a combination of multiple therapies.^76,233^ The benefits and intensity of antimicrobial nanoparticles compared to chemical antibiotics are discussed in Tables 7 and 8, respectively.

**Table 7:** Difference between nanoantibiotics and chemical antibiotics

Chemical antibiotics	Nanoantibiotics	Ref.
Antibiotics possess distinct functional groups that effectively impede the activity of biomolecules and their production	DNA and enzyme synthesis are inhibited. Generation of ROS that induces harm to cellular constituents. In addition, prevents energy transduction by interfering with the chain reaction of transmembrane electron transport and emitting heavy metal ions that induce detrimental consequences	234
Permeability loss at the membrane's niche	Transport across membranes is disrupted. Adsorbing on the surface, metal nanoparticles (*e.g.*, ZnO, Ag) generate ROS that harm cellular components (*e.g.*, cell membrane/wall)	235 and 236
The potential for antibiotic resistance exists, as bacteria can develop genes that confer resistance	Provide bacterial organisms with resistance to genetic molecules	234 and 235
High manufacturing costs and time are required	Need low manufacturing costs and times	236

**Table 8:** A comparative analysis of antimicrobial nanomaterials and antibiotics.^237^

		Intensity	
		Antibiotics	Antimicrobial nanomaterials
Production	Scale-up production	High	Low
	Purification	High	Low
	Standardization	High	Moderate
Biofilm prevention	Efficiency	Low	Moderate
	Durability	Low	High
	Permeability	Low	Low
Bacterial killing	Target	Specific	Multiple
	Killing efficiency	High	Moderate
	AMR evolution	High	Moderate
	AMR prevention	Low	Moderate
	Biodistribution	Intracell or intermembrane	Extracell/intracell/intermembrane
*In vivo* application	Controllability	Low	Moderate
	Selectivity	Moderate	Low
	Side effect	Low	Moderate

A side effect is defined as an unintended consequence of antibacterial compounds that is neither suboptimal nor complements their intended function. Controllability pertains to the capacity of antibacterial agents to undergo engineering processes that produce desired biological responses within the body, including but not limited to protracted blood circulation and targeted biodistributions.

Antibiotic discovery and development in the twentieth century had a substantial impact on the battle against bacterial illnesses. In contrast, bacterial antibiotic resistance represents a far more significant problem due to the constant evolution of bacterial genomes. In order to address this challenge, numerous domains are implementing nanotechnology, including targeted antibiotic delivery and antibacterial vaccination facilitated by nanoparticles.^238^ According to several reports, zinc oxide, silver, and gold nanoparticles work well as antimicrobial agents against bacteria that are resistant to multiple drugs, including *Escherichia coli*, MRSA, Pseudomonas, and Klebsiella. Nanoparticles present several benefits when employed as transport for antimicrobial agents. These include enhanced bioavailability, decreased probability of drug toxicity, and the ability to accumulate sub-therapeutic drugs. Furthermore, an additional promising approach that may yield positive results is nanophotothermal therapy, which makes use of fullerene and functionalized antibodies as nanostructures.^239^ However, despite its ability to aid in the battle against antibiotic-resistant bacteria, nanopreparations may be hazardous to the environment. Even though they have antibacterial action and other promising applications, it is important to consider any possible harm to the environment. In addition, the environmental and biological risks posed by nanoparticles vary. Their large surface area, compact size, and unique physicochemical properties allow them to interact with both biological matter and the surrounding environment. Ecological processes, ecosystems, and species may suffer as a result of these interactions. Therefore, in order to address the potential ecotoxicity of nano preparations, comprehensive risk assessment processes should be devised. This involves evaluating the potential risks to the environment and public health, in addition to the exposure routes and hazards. It is essential to establish regulatory frameworks that ensure the ethical and sustainable development, use, and disposal of nanopreparations.^240,241^

Several methods using drug delivery systems based on nanotechnologies, such as silica mesoporous nanoparticles, polymeric nano-vehicles, and liposomes, have been shown to be successful in combating bacterial strains that are resistant to drugs.^63,242–244^ Considering all of the useful uses for nanoparticles, scientists are now investigating possible drug delivery methods that might use nanoparticles to maximize the therapeutic usage of antibiotics while minimizing their negative effects. Table 9 shows instances of how applied nanomaterials are being used to fight drug resistance.

**Table 9:** Nanomaterial against the emergence of antibiotic resistance

Name of nanomaterials	Applications	Ref.
Polymeric nanoparticles	Overcoming cancer cells' resistance to many drugs with combination treatment	245
Metal-based nanoparticles	Enhancing the efficacy of antimicrobial agents against resistant pathogens	246
Gold nanoparticles	Treating chemotherapy-associated multidrug resistance	247
Lipid-based nanoparticles	Preventing bacterial resistance through targeted administration of antibiotics	248
Quantum dots	Pharmaceutical personalized medicine through imaging and monitoring of drug-resistant cancer cells	249
Mesoporous silica nanoparticles	Controlled release of antibiotics to combat bacterial resistance	250
Nano emulsions	Antimicrobial drugs delivered specifically to combat resistant infections	251
Dendrimers	Delivering gene-silencing drugs to fight medication resistance in viral illnesses	252

The high therapeutic index and effectiveness of nanotechnology against microorganisms make it a potential therapeutic approach.^63^ For the majority of bacterial infections, particularly those involving pathogens resistant to several drugs, nanoparticles provide a promising substitute. Antibiotics and nanoparticles work very well together when paired, either alone or in combination. Promising approaches include nanomaterials that may support improved medication delivery and release while also responding to a variety of endogenous and external stimuli to kill microorganisms.^64,253^

## 7. Safety and efficacy of nanomaterials

Antimicrobial nanomaterials have recently been the subject of much-increased research with the goal of treating biofilm infections and multidrug-resistant (MDR) planktonic bacteria. The creation of appropriate *in vivo* and *in vitro* models that accurately reflect the safety and efficacy of nanoparticles is necessary to provide clinical feasibility for their use.^254^ Antimicrobial Ag NPs make up the majority of formulations that are presently in clinical trials. Although NPs can potentially treat bacterial infections, a number of obstacles need to be overcome before they can be effectively applied in the clinic. These include further research into how NPs interact with different cells, tissues, and organs, the best dosage, appropriate delivery methods, and toxicity following both acute and extended exposure.^255,256^ Despite the fact that nanoparticles (NPs) have demonstrated promise in antimicrobial treatment, there are several drawbacks to using them.

Toxicity: Certain NP forms may be harmful to cells, resulting in unfavorable side effects. NP toxicity can be impacted by surface charge, shape, and size.^255^

Difficulty in targeting particular cells: It limits the efficiency of NPs and raises the possibility of adverse effects. It can be difficult to target certain cells or tissues in the body.

Ineffective against some bacteria: Treatment may be more difficult when using NPs since they may be less efficient against certain bacterial species or bacterial biofilms.^257^

Potential for resistance: It is feared that similar to how bacteria become resistant to antibiotics, they may also become resistant to NPs. This may restrict the long-term efficacy of antibacterial medicines based on NPs.^255,258^

Regulatory obstacles: As NPs are a relatively new field of study in antimicrobial treatment, getting regulatory permission for their usage may be difficult. Their usage and availability in therapeutic settings may be restricted as a result.^259^

Cost: Because NPs can be costly to create and produce, some patients may not be able to obtain or afford them.

However, in contrast to conventional antibiotics, the distinctive physical structure of NPs confers a number of substantial advantages. The advantageous effects and negative consequences of nanomaterials in therapeutic usage are specified in Table 10.

**Table 10:** Advantages and disadvantages of nanomaterials in therapeutic usage

Advantageous effects	Negative consequences
Enhanced solubility and regulated medication release	
Extended duration of therapeutic effects due to the gradual elimination process	Long-term systemic exposure enhances the therapeutic effect of medications delivered locally
A wide variety of therapeutic efficacy	Organs and metabolic systems exposed to nanotoxicity
Less chance of bacterial resistance emerging	Intravascular nanoparticles are deposited in the body's cells and organs
The capacity to cross biological barriers, such as the blood–brain barrier	
Low level of sensitivity	Inadequate accessibility to characterization techniques that are independent of nanoparticle properties
Relatively little side effects as compared to chemical antimicrobials	
Accumulation of medications for the purpose of delivering them to specific sites	

## 8. Plant-based nanomaterials as a viable substitute for multidrug-resistant bacterial infections

Plant-based nanoparticles for treating MDR-bacterial infections are produced by the secondary metabolism of plant tissues, such as roots, flowers, bark, stems, shoots, seeds, and leaves. These secondary metabolisms react very quickly when exposed to external stimuli and other signals.^260^ The biosynthesis of metal nanoparticles (NPs) aroused interest in the clarification and characterization of the processes of metal ion absorption and bioreduction by plants due to the great stability and quick pace of biosynthesis of plant-based NPs. Numerous studies have demonstrated that plant extracts contain a wide range of secondary metabolites, including coenzymes that function as reducing and stabilizing agents in the bioreduction reaction that produces metallic NPs, as well as phenolic compounds, alkaloids, flavonoids, tannins, saponins, steroids, terpenoids, *etc.* These metabolites can act as potential precursors for the safe synthesis of nanomaterials.

**Fig. 10:**
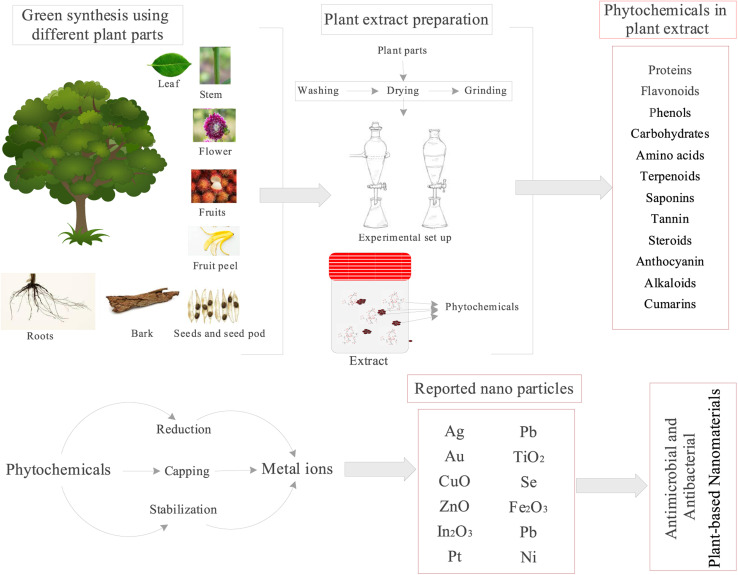
Different parts of the plant are used for extract and mechanism of formation of nanoparticles.

In contrast to AgNPs used alone, Jyoti *et al.* (2016) demonstrate that ‘green’ AgNPs, combined with conventional antibiotics, have an advantageous synergistic effect.^261^ The study revealed that the aqueous extract of *Urtica dioica* (Urticaceae) leaves capped AgNPs, and amoxicillin had a synergistic enhancing role. The maximum impact was reported for amoxicillin with easy bottom-up ‘green’ generated AgNPs against *Serratia marcescens*, with a 17.8-fold increase in the inhibitory zone.^261^ Antibiotics and plant-based nanoparticles (NPs) have been found to interact in additional ways, including AgNPs and *Zea mays* extract (Poaceae) from corn leaf detritus. AgNPs and *Typha angustifolia* leaf extract (Typhaceae), combined with gentamicin, cefotaxime, and meropenem, demonstrated efficacy against *E. coli* and *Klebsiella*. Kanamycin and rifampicin were efficacious against five strains of bacteria.^262^ Thus, antibiotics and synthetic green metallic nanoparticles seem to work well together to reduce toxicity and resistance in multidrug-resistant bacterial infections.^263^ The green synthesis of metal NPs is shown in Fig. 10.

## 9. Extraction of plant extract

Extracts from a wide variety of plant species have already been applied to the biosynthesis of NPs, with success, including cobalt, copper, silver, gold, palladium, platinum, zinc oxide, and magnetite.^260^ Plant extracts are made by drying and treating plant parts such as leaves, roots, flowers, fruits, peel, seeds, bark, and stems with methanol.^264^ Plant extracts contain various phytochemicals such as proteins, flavonoids, phenols, carbohydrates, amino acids, terpenoids, saponins, tannins, steroids, anthocyanin, alkaloids, and coumarins, which carry out the reduction, capping, and stabilizing processes on the metals added to the extract, resulting in the synthesis of green metal NPs.^265^

Plants such as *Aspalathus linearis*, *Caesalpinia spinosa*, *Centella asiatic*, *Cinnamomum cassia*, *Citrus unshiu*, *Coffea canephora*, *Myrica Esculenta*, and *Vitis vinifera* produce aspalathin, tannic acid, kaempferol, cinnamic acid, narirutin, chlorogenic acid, myricetin, and resveratrol phytochemical compounds respectively for the synthesis of AuNPs and *Centella asiatic*, *Cinnamomum zeylanicum*, *Cinnamomum zeylanicumverum*, *Citrus paradise*, *Curcuma longa*, *Cyclopia intermedia*, *Cynomorium coccineum*, *Eucalyptus globus*, *Memecylon umbellatum*, *Mentha pulegium*, *Stachys tuberifera*, *Thymus vulgaris*, and *Vitis vinifera*, which further produce quercetin, cinnamaldehyde, eugenol, naringin, curcumin, hesperidin, gallic acid, caffeic acid, 4 *N*-methyl benzoic acid, diosmin, stachyose, thymol, and resveratrol compounds respectively for synthesis of Ag NPs. Moreover, Aspalathin and ellagic acid extracted from *Aspalathus linearis* and *Rubus idaeus* are used to produce RhNP and ZnNP, respectively.^266^

In addition to plant extracts, live plants can synthesize metal nanoparticles. When silver is present in the substrate, some plants, such as *Brassica juncea* (Mustard greens), *Medicago sativa* (Alfalfa), and *Helianthus annuus* (Sunflower), can accumulate a significant amount of silver. In addition, Ni, Co, Zn, and Cu NPs have been synthesized in living plants. The bioaccumulated metals are stored in various parts of the plants, and metal NPs are obtained directly from these parts through chemical processes.^267^

### 9.1. Types of phytochemicals in the extract and their role

Proteins, flavonoids, phenols, carbohydrates, amino acids, terpenoids, saponins, tannins, steroids, anthocyanin, alkaloids, and coumarins are some of the most common phytochemicals used in the production of metal NPs. These phytochemicals reduce, cap, and stabilize metal nanoparticles (NPs). For example, polysaccharides, vitamins, amino acids, proteins, phenolics, saponins, alkaloids, and/or terpenes are used to reduce and stabilize the Ag ions in AgNPs.^268^ In producing gold NPs, various chemical moieties in biogenic complexes act as reducing agents, reducing gold metal ions and forming nanoparticles. According to some studies, biomolecules such as flavonoids, phenols, proteins, and others are essential in lowering metal ions and topping gold nanoparticles in plant extracts.^269^ Aromatic amines can also produce Pt and Pd-based NPs *via* domino and tandem reactions.^270^

Metal NPs (such as Au, Ag, Cu, Pd, Se, and Pt NPs) are produced by amino acids such as -amylase, urease, and protease by binding metal ions to exposed free cysteine and reducing the metal ion. The reduced metal ions are then capped by the formation of disulfide bonds with the help of the lysosome enzyme. Polyphenols such as flavonoids, phenolic acid, terpenoids, and proteins reduce and stabilize metal NPs *via* a similar mechanism^271^—the metal-chelating ability and, thus, the metal-reducing ability of phenolic acid's nucleophilic aromatic rings. During bioreduction, the –OH groups of flavonoids (quercetin and myricetin) and terpenoids combine with metal ions and are oxidized to carbonyl groups. Proteins aid in stabilising metal NPs during the nucleation process, and capping the nucleated metal NPs aids in their stability.^272^

### 9.2. Various parameters of extract affecting morphology and size of particles

Factors influencing the preparation and properties of plant-based metal NPs include the type of plant extract used, its concentration, the pH of the medium, the concentration of the metal salt, contact time, and temperature. These variables have been shown to influence the rate, properties, and quantity of prepared nanoparticles. Makarov *et al.* (2014) observed that the size and shape of the NPs are highly dependent on their location. The most common plant extract parameters influencing particle morphology, shape, and size are described below.^268^

✓ Plant extract type and concentration: The concentration of plant biomass/extract often determines the efficiency of nanoparticle synthesis. Several researchers discovered that increasing the biomass dosage boosts nanoparticle production and changes the shape of nanoparticles.^273^ Chandran *et al.* (2006) used Aloe vera leaf extract to modulate the shape and size of the synthesized Au nanoparticles.^274^ Most of the Au nanoparticles were triangular and ranged in size from 50 to 350 nm, depending on the extract used. Adding small amounts of the extract to the HAuCl_4_ solution resulted in the formation of larger nanogold triangles. Furthermore, as the amount of extract increased, the ratio of nanotriangles to spherical nanoparticles decreased.^273^

✓ pH: A solution's pH influences nanoparticle synthesis using green technology methods. According to the researchers, the solution medium's pH affects the synthesised nanoparticle's size and texture. This is caused by the formation of nucleation centers, which increases as pH increases.^273^ As a result, the size of nanoparticles can be controlled by adjusting the pH of the solution media. Soni and Prakash (2011) demonstrated the effect of pH on the shape and size of the synthesized silver nanoparticle.^275^

✓ Temperature: In most cases, using green technology to synthesize nanoparticles requires temperatures less than 100 °C or ambient temperature. The nature and size of the nanoparticle formed are determined by the temperature of the reaction medium.^276^

✓ Pressure: The shape and size of the synthesized nanoparticles are affected by the pressure applied to the reaction medium. The rate of metal ion reduction using biological agents was discovered to be much faster at ambient pressure conditions.^277^

✓ Time: The time and the incubated reaction medium significantly impact the quality and type of nanoparticles synthesized using green technology.^278^ Variations in time can occur in various ways, including particle aggregation because of long-term storage; particles may shrink or grow due to long-term storage; they may have a shelf life, and so on, all of which affect their potential.

✓ Environment: The surrounding environment significantly impacts the nature of the synthesized nanoparticles. In many environments, a single nanoparticle quickly transforms into core–shell nanoparticles by absorbing materials or reacting with other environmental materials *via* oxidation or corrosion.^279^ In a biological system, the nanoparticles form a coating that thickens and expands them. Furthermore, the environment influences the physical structure and chemistry of the synthesized nanoparticles. There are a few examples of how the environment affects the nature of synthesized nanoparticles. When the environment of the zinc sulfide nanoparticles was changed from wet to dry, the crystalline nature of the nanoparticles changed immediately. Similarly, the chemical nature of cerium nitrate nanoparticles changes depending on the amount of peroxide present in the solution in which they are suspended.

✓ Proximity: When individual or isolated nanoparticles meet or near the surface of another nanoparticle, their properties are altered in most cases. This changing behavior of nanoparticles can be used to create more tailored nanoparticles. The proximity effect of nanoparticles has many implications, including particle charging, substrate interactions, and magnetic properties of the nanoparticles.^280^

✓ Other considerations: Secondary metabolites are abundant in various living systems, including plants, and act as reducing and stabilizing agents in the synthesis of nanoparticles. However, the composition of these metabolites varies depending on the type of plant, plant part, and extraction method used. The concentration of metal ions also significantly impacts the effectiveness of various phytochemicals, which in turn determines the morphology and size of the metal NPs produced.^281^

### 9.3. Literature reports on different parts of plants used as an extract for the synthesis of NPs

AgNPs and AuNPs are the most investigated plant-based NPs, primarily associated with developing potent antimicrobial NPs. CuO, ZnO, Fe, Pt, Pd, Pb, Ni, In_2_O_3_, TiO_2_, and Se NPs are other metal NPs produced from plants for antimicrobial activities. Plant parts such as leaf, roots, stem, fruit, flower, seed, stem, bark, peel, and tuber synthesize metal NPs.^282^ The details of various plant parts of different plants that are used for the synthesis of metal NPs are discussed in Table 11.

**Table 11:** Different parts of various plants used in the synthesis of metal nanoparticles

NPs	Plant parts	Scientific names	References
Ag	Leaf	*Acalypha indica*, *Alstonia scholars*, *Achras sapota*, *Arbutus unedo*, *Azhadirachta indica*, *Bixa orellana*, *Catharanthus roseus*, *Cinnamomum camphora*, *Coleus aromaticus*, *Coriandrum sativum*, *Citrus limon*, *Calotropis gigantia*, *Ceratonia siliqua*, *Cassia auriculata*, *Cephalandra indica*, *Desmodium triflorum*, *Dalbergia sissoo*, *Euphorbia hirta*, *Elaeagnus indica*, *Elaeagnus latifolia*, *Ficus carica*, *Ficus religiosa*, *Gliricidia sepium*, *Lippia citriodora*, *Parthenium*, *Paederia foetida*, *Phyllanthus amarus*, *Hevea brasiliensis*, *Musa paradisiaca*, *Nerium oleander*, *Ocimum bacillicum*, *Odina wodier*, *Juglans regia*, *Phyllanthus reticulates*, *Brillantaisia owariensis*, *Crossopteryx febrifuga*, *Senna siamea*, *Urtica dioica*, *Zea mays*, and *Typha angustifolia*	267, 282, 283 and 284
	Roots	*Portulaca oleracea*, *Macrotyloma uniflorum*, *Glycyrrhiza glabra*, *Cochlospermum religiosum*, *Boswellia ovalifoliolata*, *Thespesia populnea*, *Svensonia hyderobadensis*, *Shorea tumbuggaia*, and *Catharanthus roseus*	282
	Stem	*Portulaca oleracea*, *Macrotyloma uniflorum*, *Glycyrrhiza glabra*, *Cochlospermum religiosum*, *Boswellia ovalifoliolata*, *Thespesia populnea*, *Svensonia hyderobadensis*, and *Shorea tumbuggaia*	
	Fruit	*Solanum xanthocarpum*, *Vitis vinifera*, *Solanum trilobatum*, *Carambola* sp., *Carica papaya*, *Momordica cymbalaria*, *Piper longum*, and *Lycopersicon esculentum*	
	Flower	*Ipomoea indica*, *Mirabilis jalapa*, *Plumeria alba*, *Millingtonia hortensis*, *Cassia auriculata*, *Carthamus tinctorius*, *Gnidia glauca*, and *Boswellia serrate*	
	Fruit peel	*Musa* sp	
	Seed	*Brassica nigra*, *Glycine max*, *Cyperus esculentus*, *Butyrospermum paradoxum*, and *Syzygium cumini*	
	Tuber	*Curcuma longa* and *Dioscorea bulbifera*	267
Au	Leaf	*Abutilon indicum*, *Acalypha indica*, *Aloe vera*, *Amaranthus spinosus*, *Argemone mexicana*, *Azadirachta indica* L., *Bacopa monnieri*, *Bougainvillea glabra*, *Butea monosperma*, *Cacumen platycladi*, *Cicer arietinum* L., *Cinnamomum zeylanicum*, *Costus igneus*, *Diospyros ferrea*, *Erythrina variegate*, *Euphorbia hirta*, *Ficus benghalensis*, *Geranium* sp., *Gymnema sylvestre*, *Hibiscus rosa-sinensis*, *Hibiscus sabdariffa*, *Hygrophila spinosa*, *Ipomoea carnea*, *Magnolia kobus*, *Diopyros kaki*, *Mangifera indica*, *Mimosa pudica*, *Nepenthes khasiana*, *Nerium oleander*, *Ocimum sanctum*, *Olea europaea*, *Padina gymnospora*, *Phoenix dactylifera*, *Salix alba*, *Sesbania grandiflora*, *Silybum marianum*, *Spinacia oleracea*, *Suaeda monoica*, *Terminalia arjuna*, *Terminalia catappa*, *Vitis vinifera*, and *Piper guineense*	282 and 285
	Stem	*Ipomoea carnea*, *Hibiscus sabdariffa*, and *Salvadora persica*	282
	Root	*Ipomoea carnea*, *Morinda citrifolia*, *Coleus forskohlii*, *Morinda citrifolia* L., and *Panicum maximum*	
	Bark	*Eucommia ulmoides*, *Acacia nilotica*, *Ficus religiosa*, and *Cassia fistula*	
	Fruit	*Averrhoa bilimbi*, *Lansium domesticum*, *Genipa americana*, *Citrus limon*, *Citrus reticulate*, *Citrus sinensis*, *Citrus maxima*, *Terminalia arjuna*, and *Nitraria schoberi*	
	Fruit peel	*Punica granatum*	
	Flower	*Tagetes erecta*, *Moringa oleifera*, *Cassia auriculata*, *Ixora coccinea*, *Nyctanthes arbor-tristis*, *Bauhinia purpurea*, *Plumeria alba*, and *Gnidia glauca*	
	Seed	*Cajanus cajan*, *Abelmoschus esculentus*, and *Vitis vinifera*	
	Seed pod	*Elettaria cardamomum*	
ZnO	Leaf	*Pongamia pinnata*, *Solanum nigrum*, *Eichhornia crassipes*, *Ocimum basilicum*, *Anisochilus carnosus*, *Azadirachta indica*, *Aloe vera*, *Agathosma betulina*, *Phyllanthus niruri*, *Moringa oleifera*, *Plectranthus amboinicus*, *Santalum album*, *Vitex negundo*, *Parthenium hysterophorus*, *Calatropis gigantean*, and *Sphathodea campanulata*	
	Flower	*Trifolium pretense* and *Vitex negundo*	
	Fruit	*Rosa canina*	
	Fruit peel	*Nephelium lappaceum*	
	Rhizome	*Coptidis rhizoma*	
In_2_O_3_		*Aloe vera*	
Pt	Leaf	*Diopyros kaki* and *Anacardium occidentale*	
Pb		*Jatropha curcas*	
CuO	Leaf	*Phyllanthus reticulates* and *Erigeron bonariensis*	286
TiO_2_	Leaf	*Catharanthus roseus*, *Syzygium aromaticum*, *Elettaria cardamomum*, *Cinnamomum verum*, *Withania somnifera*, *Eclipta prostrate*, *Glycyrrhiza glabra*, and*Ledebouria revolute*	287 and 288
Se	Leaf	*Capsicum annuum* and *Urtica dioica*	289
Fe	Leaf	*Terminalia chebula*, *Hordeum vulgare*, *Rumex acetosa*, *Ruellia tuberose*, *Tridax procumbens*, *Gardenia jasminoides*, *Lawsonia inermis*, *Moringa oleifera*; *Ageratum conyzoides*, *Eichhornia crassipes*, *Eichhornia crassipes*, *Anacardium occidentale*, *Cynometra ramiflora*, and *Lantana camara*	290 and 291
	Bran	*Sorghum bicolour*	282
	Root	*Ageratum conyzoides*	292
	Fruit peel	*Garcinia* sp., *Mangostana* sp., and *Punica granatum*	293
	Fruit	*Couroupita guianensis*	282
	Flower	*Callistemon viminalis*	294
Pd	Peel	*Annona squamosa* and *Piper betle*	282
	Leaf	*Pulicaria glutinosa*, *Pulicaria glutinosa*, and *Terminalia chebula*	
	Fruit	*Vitis vinifera*	267
Ni	Leaf	*Ocimum tenuiflorum*	

Plant leaves are the most common parts used in biosynthesis metal NPs. Leaf of various plants such as *Acalypha indica*, *Alstonia scholars*, *Achras sapota*, *Arbutus unedo*, *Azhadirachta indica*, *Bixa orellana*, *Catharanthus roseus*, *Cinnamomum camphora*, *Coleus aromaticus*, *Coriandrum sativum*, *Citrus limon*, *Calotropis gigantia*, *Ceratonia siliqua*, *Cassia auriculata*, *Cephalandra indica*, *Desmodium triflorum*, *Dalbergia sissoo*, *Euphorbia hirta*, *Elaeagnus indica*, *Elaeagnus latifolia*, *Ficus carica*, *Ficus religiosa*, *Gliricidia sepium*, *Lippia citriodora*, *Parthenium*, *Paederia foetida*, *Phyllanthus amarus*, *Hevea brasiliensis*, *Musa paradisiaca*, *Nerium oleander*, *Ocimum bacillicum*, *Odina wodier*, *Juglans regia*, *Phyllanthus reticulates*, *Brillantaisia owariensis*, *Crossopteryx febrifuga*, *Senna siamea*, *Urtica dioica*, *Zea mays*, and *Typha angustifolia* are used in the synthesis of AgNPs whereas leaf of *Abutilon indicum*, *Acalypha indica*, *Aloe vera*, *Amaranthus spinosus*, *Argemone mexicana*, *Azadirachta indica* L., *Bacopa monnieri*, *Bougainvillea glabra*, *Butea monosperma*, *Cacumen platycladi*, *Cicer arietinum* L., *Cinnamomum zeylanicum*, *Costus igneus*, *Diospyros ferrea*, *Erythrina variegate*, *Euphorbia hirta*, *Ficus benghalensis*, *Geranium* sp., *Gymnema sylvestre*, *Hibiscus rosa-sinensis*, *Hibiscus sabdariffa*, *Hygrophila spinosa*, *Ipomoea carnea*, *Magnolia kobus*, *Diopyros kaki*, *Mangifera indica*, *Mimosa pudica*, *Nepenthes khasiana*, *Nerium oleander*, *Ocimum sanctum*, *Olea europaea*, *Padina gymnospora*, *Phoenix dactylifera*, *Salix alba*, *Sesbania grandiflora*, *Silybum marianum*, *Spinacia oleracea*, *Suaeda monoica*, *Terminalia arjuna*, *Terminalia catappa*, *Vitis vinifera*, and *Piper guineense* are used in the synthesis of AuNPs.^261,267,282–284^

Even the roots of *Portulaca oleracea*, *Macrotyloma uniflorum*, *Glycyrrhiza glabra*, *Cochlospermum religiosum*, *Boswellia ovalifoliolata*, *Thespesia populnea*, *Svensonia hyderobadensis*, *Shorea tumbuggaia*, and *Catharanthus roseus* and stem of *Portulaca oleracea*, *Macrotyloma uniflorum*, *Glycyrrhiza glabra*, *Cochlospermum religiosum*, *Boswellia ovalifoliolata*, *Thespesia populnea*, *Svensonia hyderobadensis*, and *Shorea tumbuggaia* used for production of AgNP show an extensive range plants. Various plants' fruits, flowers, and seeds are also used to synthesize AgNP. In addition to the fruit, flower, and stem, the barks of *Eucommia ulmoides*, *Acacia nilotica*, *Ficus religiosa*, and *Cassia fistula* synthesize AuNPs.^282^ Even the rhizomes of *Coptidis* sp., Bran of *Sorghum bicolour*, and coconut water are also used to synthesize the metal NPs. Fruit extracts of *Vitis vinifera*, *Couroupita guianensis*, and *Rosa canina* help synthesize Pd, Fe, and ZnO metal NPs. Thus, every part of a plant can be used to synthesize metal NPs.

## 10. Challenges and future perspective

There is a growing interest in improving drug delivery for disease resolution. As a result, candidates such as biogenic metal-based nanoparticles with improved biodistribution and pharmacokinetics have a one-of-a-kind opportunity.^295^ Metallic nanoparticles inspired by nature represent a new generation of innovative nanomedicines that mimic natural circulatory cells. These materials have been discovered to have the ability to increase blood circulation time and improve drug distribution to cells and tissues.^223^ The role of nanotechnology in the precise treatment of diseases, which often has less life-threatening side effects, has the potential to contribute to a positive shift in clinical practice toward life-saving approaches. However, their immunogenicity, scale-up, and characterization remain significant challenges during clinical trials.^296^ Aside from scaling-up issues, government regulations and overall cost-effectiveness compared to currently available chemotherapies are substantial barriers to the success of nanomedicines. The often-complex architectural design of many biogenic metal NPs may also make it challenging to perform reproducible, safe, and sufficient sample preparations. One of the most difficult challenges has been reproducibility, as minor changes in NPs size, shape, and/or surface chemistry can dramatically affect their stability, interaction with biological media, and biodistribution.^265^ As a result, reproducible nanoparticles require reliable and standardized methodologies.

Furthermore, for these materials to be excellent biological tools, the gap between the laboratory, where innovative materials are designed, and the industrial replication of the process, where reproducible preparation and manufacturing processes are carried out, must be narrowed. There is an exciting future for NPs in combining them to act as ‘nanomachines’: their mode of interaction would have a non-linear dependence on changed parameters such as temperature or pH. This approach appears to be working, and the future of nanotechnology is getting closer; however, its potential threats must be considered, and the effects of new procedures must be carefully examined before they are implemented. For example, the toxicity of nanoparticles (NPs) is critically discussed to identify potential issues before they are widely used in medicine. On the other hand, synthesis mediated by plant extracts is said to be environmentally friendly. One of the most notable trends in the synthesis of metallic NPs from plant extracts could be a beneficial strategy for characterizing the mode of action of NPs.^267^ It is a controlled synthesis that can be easily transferred to a large scale and ensures environmental safety while reducing the process's environmental impact. Taken as a whole, the use of plant-based NPs has demonstrated a variety of benefits and applications in medicine and the pharmaceutical industry. More specifically, studies show that NPs can exert antimicrobial properties alone or in combination with antibiotics, reducing the current problem of acquired resistance caused by antibiotic overuse or misuse. Given this potential application, future research should concentrate on two areas: assessing the safety aspects of using plant-based NPs (toxicity to human health and environmental safety) and reducing the environmental impact of their synthesis. More detailed development of synthesis procedures is required, as is research into the action mechanisms that mediate the antibacterial effect of NPs to make plant-based NPs a viable strategy capable of meeting society's demand for an effective solution to antibiotic resistance.

## 11. Summary

This review meticulously summarized the various emerging nanomaterial platforms to mitigate antimicrobial resistance. The review includes the synthesis of nanomaterials the extraction of cost-effective and eco-friendly plant-based nanomaterials as a viable alternative to combat multidrug-resistant bacterial infections. In addition, this study examines the toxicity of nanomaterials by thoroughly investigating the physicochemical properties of these materials, intending to utilize them as a feasible alternative to chemical antibiotics. The mechanism of action of nanomaterial on pathogenic organisms is also elaborately presented in this review, along with the techniques of different nanomaterials to fight against microbial pathogens. Moreover, applications of nanomaterials in medical science, novel drug delivery systems for nanomaterials, and the advantages of antimicrobial nanomaterials over chemical antibiotics are also covered in this review. Therefore, this review could be valuable in establishing innovative and effective therapeutic approaches for antimicrobial therapy. It is evident that nanomaterials have the potential to bring about a revolutionary change in medical science by mitigating antimicrobial resistance in the human body, and they can serve as a substitute for conventional chemical antibiotics. Plant-based nanomaterials can be crucial in reducing toxicity in the human body and ensuring human safety, offering both environmental friendliness and economic benefits. In addition, nanomaterials have the potential to have a significant impact on novel drug delivery methods and therapeutic applications.

## Author contributions

Sazedur Rahman: writing – original draft, data curation, investigation, visualization. writing – review & editing, visualization. Somya Sadaf: writing – original draft, data curation, investigation, visualization. writing – review & editing, visualization. Md Enamul Hoque: conceptualization, formal analysis, investigation, data curation, writing – review & editing. Akash Mishra: writing – review & editing. Nabisab Mujawar Mubarak: writing – review & editing, formal anaylysis. Guilherme Malafaia: formal analysis, writing – review & editing. Jagpreet Singh: conceptualization, formal analysis, investigation, data curation, writing – review & editing.

## Conflicts of interest

We confirm that there are no known conflicts of interest associated with this work, and there has been no significant financial support for this work that could have influenced its outcome. Furthermore, we ensure that the manuscript has been read and approved by all named authors and that there are no other persons who satisfied the criteria for authorship but are not listed. We further confirm that we have all approved the authors' order listed in the manuscript. Due care has been taken to ensure the integrity of the work.

## Supplementary Material
